# A dynamic model of nonviolent resistance strategy

**DOI:** 10.1371/journal.pone.0269976

**Published:** 2022-07-27

**Authors:** Erica Chenoweth, Andrew Hocking, Zoe Marks

**Affiliations:** 1 John F. Kennedy School of Government, Harvard University, Cambridge, Massachusetts, United States of America; 2 Independent Scholar, Ithaca, New York, United States of America; University of Hong Kong, HONG KONG

## Abstract

Why have some nonviolent revolutions succeeded even with modest participation numbers, while others have failed despite massive mobilization? We develop an agent-based model that predicts the outcomes of three well-known activism strategies. The first rapidly recruits a wide number of activists, which overwhelms the opponent’s support network and encourages large-scale defections. In the second, activists who have already mobilized remain committed to success and inspire other civilians to protest even when they are unable to protest themselves. In the third strategy, campaigns focus their energy and influence directly on the regime’s pillars of support. We find that this third strategy outperforms the others in generating defections, even when the size of the campaign is small. When activists have information about pillars’ levels of loyalty to the regime, they can target persuasion on the pillars most likely to defect. Importantly, for small or medium-sized movements, the strategy of focusing on pillars—especially the least loyal pillars—is more likely to yield success than relying on rapid mobilization and numerical advantage alone.

## Introduction

Prior research on people power revolutions has found that participation size is a powerful correlate of campaign success. Numerous studies argue that when people rise up in large numbers against their governments, movements are more likely to win [1–4]. Moreover, movement momentum—increasing numbers of events in quick succession—can help smaller campaigns influence large-scale political outcomes [5]. Such studies speculate that movement momentum is particularly impactful because it increases pressure on security forces to defect from the government [6]. For instance, in Sudan, a revolutionary movement in 2019 rapidly mobilized mass demonstrations, sit-ins, and strikes to force the ouster of dictator Omar al-Bashir, who was deposed by his own military. Defection of key political and societal pillars away from the power-holder is a key mechanism through which mass movements achieve change [1]. Movements often use a variety of tactics to induce defections, including protests, strikes, boycotts, and other forms of public pressure [6, 7]. Such tactics can put direct pressure on pillars to stop cooperating with the authorities, and often involve making public or private personal appeals, creating financial pressure on businesses by organizing labor actions, consumer boycotts, or raising awareness among shareholders and employees, or appealing to shared norms such as patriotism, professionalism, duty, family obligation, gender norms, religious principles, and other moral codes to induce defections.

The emphasis on movement size and protest momentum alone as the pathways for achieving elite network defections has two limitations. First, cross-national observational data suggest that campaign size is a more unreliable predictor of success than previously understood, particularly in contemporary mass movements [8]. Many campaigns fail despite achieving large-scale participation during peak events. In Bahrain in 2011, for example, a popular uprising mobilized up to 7 percent of the population against the ruling monarchy, but the movement was still defeated [9]. In Iraq, over 1 million people—over 8 percent of the population—reportedly protested to topple Saddam Hussein after his military’s withdrawal from Kuwait in 1991, yet this campaign also did not achieve its demands. Conversely, some campaigns have succeeded with a relatively modest proportion of popular mobilization, such as Mongolia’s 1989–1990 pro-democracy movement, which mobilized less than 1 percent of the population and yet succeeded.

Second, existing observational research does not account for whether campaigns adopt particular strategies that link mass mobilization to defections. We also lack systematic evidence on the comparative advantages of adopting such strategies, compared to mass mobilization alone. Instead, popular discourse about such movements implies that mass mobilization is often spontaneous and leaderless, and that large-scale mobilization often occurs without an organized strategy to influence or weaken the opponent’s pillars of support. An alternative perspective common in sociological studies, is that organizational features, resource mobilization, and strategic choices are all paramount [10]. Yet we are not aware of many studies that use computational modeling to systematically evaluate the ultimate outcomes of different strategies of nonviolent mass movements.

This article seeks to address these two issues with a computational model that varies participation thresholds, as well as the strategies used by activists to influence the balance of political power. The scope of our study builds upon and extends existing computational models that address thresholds for revolutionary success but focus on armed revolutions. The parameters for successful revolution in these models are neither practical nor desirable because they involve killing a vast number of the movement’s opponents [11, 12]. While such models have yielded important insights regarding the dynamics of violent revolution, far more mass revolutionary movements in the contemporary era—including the 2010–2011 Arab Spring uprisings in Tunisia, Egypt, Bahrain, and Yemen—have relied primarily on unarmed, nonviolent mobilization rather than on armed insurrection or guerrilla warfare, even when they faced brutally violent regimes [13]. Consequently, model parameters that require the killing or armed capture of state opponents and security forces have less relevance to contemporary revolutions than parameters that require nonviolent defections by pillars of support within the opponent regime.

We present a computational model focused on the empirical phenomenon of nonviolent resistance over the past five decades, validated against all 110 mass nonviolent mobilizations from 1945–2014 that aimed to overthrow incumbent national governments. In doing so, we draw on a promising trend of using historical data to calibrate the model’s parameters [14–16]. The model accounts for and compares strategies focused on influencing the target regime’s pillars of support, as well as the rapid mobilization of fellow civilians and activists. Although our model does not directly account for potential strategies the regime might use to prevent defections—an issue we address in the conclusion—our results do suggest that strategies that close the social distance between activists and the regime’s pillars of support lead to a higher chance of success than relying on mass mobilization alone.

Taken together, these observations indicate the importance of accurate information about the opposition, focused disruptions among the opponent’s pillars of support, rapid mobilization of civilian participants, and a sustainable activist base for generating movement success. These findings shed light on why some nonviolent resistance campaigns succeed even though they are relatively small in size, whereas others fail despite large-scale mass mobilization.

## Study design and data

An agent-based model enables us to look at change over time in individual and categorical (i.e. group-level) behavior according to different sets of rules (parameters) that reflect observed features of nonviolent collective action. Agent-based modeling gives us a dynamic view of mobilization patterns and threshold effects. To engage with existing work modeling violent resistance, we adapt and expand Alessandro Moro’s agent-based model of violent revolution to incorporate distinctive features of nonviolent uprisings [12]. Moro refers to his primary agents as “citizens,” which we have modified to “civilians” in our study. Our model also introduces two new types of agents. Rather than revolutionaries who are committed to violent resistance, we include revolutionary activists who are committed to nonviolent resistance. Second, we add agents representing the regime’s pillars of support—powerful political, economic, or social elites upon whose cooperation the regime depends to maintain its grip on power. A new rule allowed these pillars and the security force agents to defect.

Our model therefore includes four types of agents typically found in contentious politics:

- **Civilians**: These make up the majority of the agents; they can decide to remain inactive or choose to join the resistance. If they do, we call them “nonviolent civilians.” Once they’ve joined, they enter a cycle of time alternating between protesting and being dormant. Depending on model parameters, they can also leave the resistance and cease protesting altogether (mimicking real-world burnout or fatigue dynamics).
- **Activists**: These agents are committed to nonviolent resistance in all circumstances. Like nonviolent civilians, they go through cycles of active protest and dormancy.
- **Pillars**: These agents represent the political, social, and economic pillars of society, whose power supports the regime. Their allegiance is necessary to maintain the status quo.
- **Police**: These are the security forces, representing army or police personnel. They arrest or kill civilians and activists, but they can also defect and stop repressing activists and nonviolent civilians.

**Fig 1:**
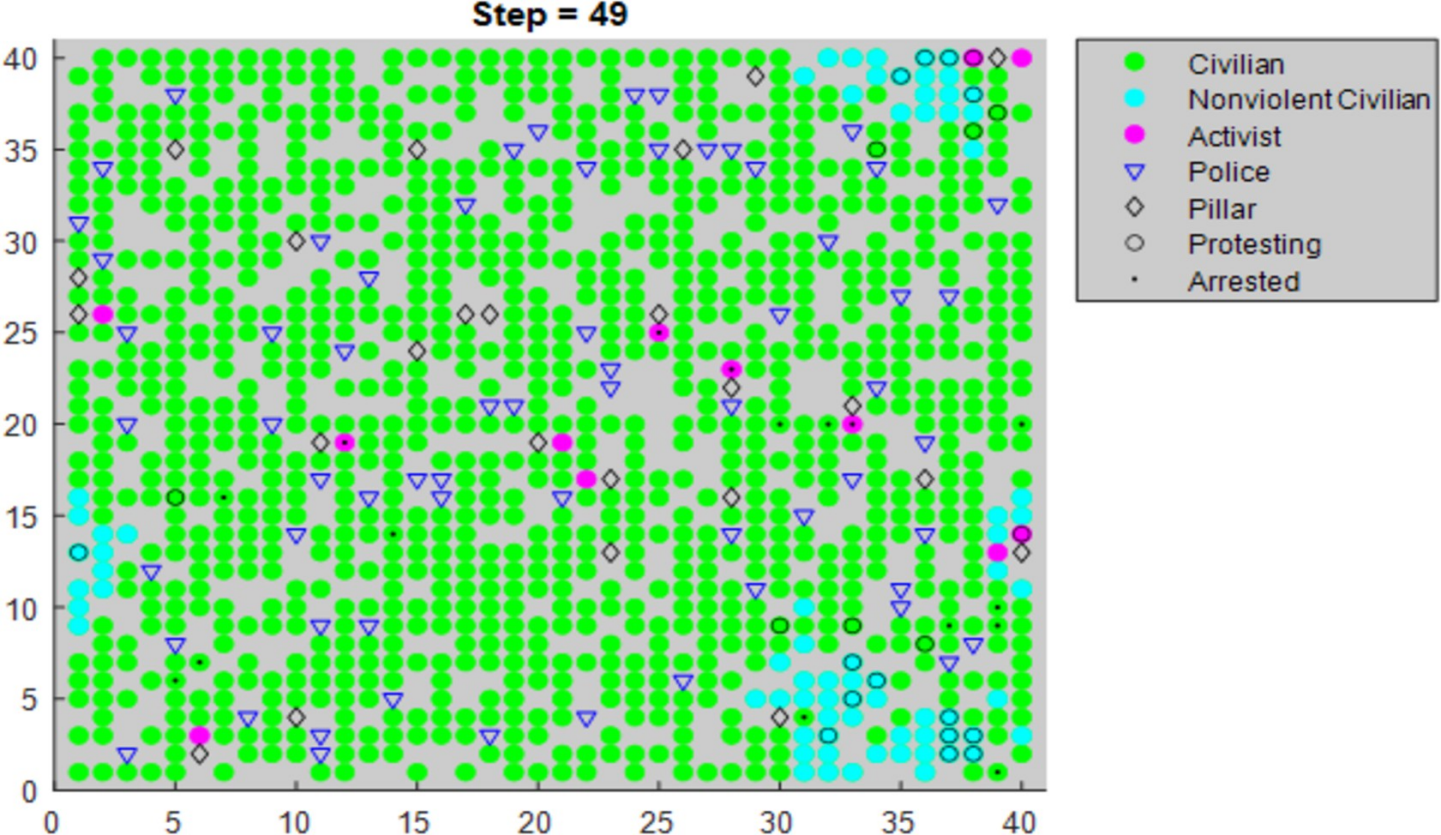
Example of resistance movement at an advanced timestep.

Following from similar work [11, 12], the model places agents on a 40x40 torus lattice (i.e. a grid in which the left edge connects with the right edge and the top connects with the bottom). All rules relate to an agent and their neighborhood. The neighborhood is a circle around the agent defined by a vision parameter of 4, meaning an agent can see any agents within a radius of four spaces around them. Fig 1 shows the lattice at a late time step in a modeling sequence simulating a large nonviolent resistance.

As mentioned above, only activists and nonviolent civilians can protest. However, civilians may have protested in a previous turn. Hence, green circles with black boundaries are civilians who started their turn by protesting (e.g. becoming “nonviolent civilians,”) but then left the resistance, ceasing to be nonviolent civilians.

While the lattice primarily represents agents in physical distances, it also pertains symbolically to social distances. A key idea fundamental to this model (and to agent-based modeling in general) is that agents or people do not necessarily respond to information about the entire society, but they respond to what they “see” and experience firsthand. For instance, if a person hears that 0.1% of the population is protesting somewhere in their nation, it does not affect them as much as encountering nearly everyone around them protest, even though the only difference is their physical proximity to that 0.1%.

### Rules

The following rules explain how the agents function.

#### Rule C: Civilians join the nonviolent resistance

The foundational action in the model is the mobilization of individual civilian agents, who can remain inactive or can join the nonviolent resistance (i.e. become nonviolent civilians). Epstein (2002) and Moro (2016) used the following rule to determine when a civilian becomes active in armed rebellion:

Grievance−Cost>afixedthreshold

G−C>f

However, it is clear from scholarship on the determinants of protest participation that social connections and protest options influence individuals’ decisions about whether to participate in mass uprisings, through both peer pressure and information cascades [17–19]. Thus, our model attempts to explicitly account for the role of social connections and information about protest opportunities in shaping civilians’ motivation to join the resistance [20–24]. Consequently, we include a peer pressure term as short-hand for these twin effects. Civilians join the resistance when:

(Grievance*PeerPressure)>(fixedthreshold+Cost)

(G*P)>(f+C)

Grievance is a value between 0 and 1, which is calculated as a function of individual hardship experienced by the agent and a dynamic value of government legitimacy. The calculation of hardship is based on a uniquely assigned income level for each agent. Modeled with an inverse logit function of the expected income in the population minus the agent’s income, the agent’s hardship ranges between 1 to 0 and decreases with higher income. This calculation is unchanged from Moro’s model [12]. Government legitimacy is a global variable. While Moro used a fixed value for government legitimacy, our model uses a starting value that can be decreased based on the actions of the policemen, which will be described below.


Grievance=Hardship*(1−GovernmentLegitimacy)

G=H*(1−GL)

In future variations of the model, each agent could have a different perceived government legitimacy based on what they currently experience, what they have experienced from the beginning of the simulation, and/or whether information spreads across social connections.

The Peer Pressure term is also a value between 0 and 1, and it is a function of the number of agents actively protesting in a civilian’s neighborhood. It is defined as:

$$Peer\;Pressure\;Term=\frac{0.5}{Peer\;Pressure\;Number}*Number\;of\;Protesting\;Agents\;in\;Neighborhood$$

$$P=\frac{0.5}{PPN}*n_{pr}$$

When the right side of the equation is larger than 1, the Peer Pressure term is set to 1. While the peer pressure term measures how much peer pressure the agent experiences, the PPN is a simulation variable set such that a larger PPN requires more protesting neighbors in order for the agent to feel the same amount of pressure.

Modified from Moro [12], the cost of rebelling is calculated as

ProbabilityofArrest*RiskAversion

(A*R)

The probability of arrest is a function of the number of policemen and protestors in the agent’s neighborhood. Using the agent types in our model:

$$A\;=\;1-exp\;(\;-w\;*\frac{n_{po}}{1+\;n_{pr}})$$

This forces A to be between 0 and 1. The variables n_po_ and n_pr_ are the number of policemen and protestors in the agent’s neighborhood. The variable w is set at 2.3 for the rule such that the probability of arrest is 0.9, i.e. 90% when only one policeman and the agent considering resisting are in the agent’s neighborhood.

Moro [12] assigned each agent a risk aversion based on their income level and the parameter determining maximum jail sentence, assuming that a wealthier individual would not risk their loss of income. We included this term, but also added an additional term in which the very poor also would not risk losing their income, which is consistent with research finding a nonmonotonic association between wealth and protest participation [25, 26].

The fixed threshold, f, is a model parameter adjusted to match model results with historical data as described below [27]. The range of values explored were slightly larger than 0, which means that Grievance * Peer Pressure needs to be slightly larger than Cost, and the value selected that best matched historical data was 0.0706. Values much larger or smaller resulted in too few or too many joining the resistance, respectively.

As noted above, Rule C is based on each agent seeing activists and nonviolent civilians protesting in their neighborhood. Only a proportion of new protesters remain highly committed, whereas others leave the resistance when they do not see others protesting. We therefore introduced a model parameter called Percent Committed, which determines what percentage of the agents join the resistance but quickly leave it as soon as protest in their neighborhood stops. In other words, the committed nonviolent civilians will join the resistance based on Rule C and remain active until they are arrested, killed, or the simulation ends. Fickle nonviolent civilians will join the resistance but leave it as soon as they do not see others participating in their neighborhoods.

#### Rule NV: Nonviolent civilians and activists protest

In Moro’s model [12], revolutionaries try to kill policemen. This rule was modified for our model in which activists and nonviolent civilians can choose to protest. Like Moro’s rule, they become visible to the security forces and are more likely to be arrested or killed when they protest.

Activists and nonviolent civilians choose to protest when the number of activists and nonviolent civilians (excluding themselves) in their neighborhood exceeds a threshold parameter called nNV.


numberofactivistsnotinjail+numberofnonviolentcivilians>protestthreshold

$$n_{act}\;+\;n_{nvc}\;>\;nNV$$

When the equation is satisfied, the agent begins to protest and their protest cycle begins. The protest cycle is the number of time steps determined by the variable Protest Cycle, which prevents agents from being in continuous protest. Agents protest for the number of time steps equal to the parameter Protest Duration, and then they do not protest for the remainder of the cycle, but their presence emboldens others to protest. When the cycle is complete, activists and nonviolent civilians protest again. If a nonviolent civilian is not committed and there are not enough nearby protesting agents, they become an ordinary civilian again and do not resume protesting, but can be activated again in future time steps. When a person leaves the resistance, the protest cycle resets. Consequently, it is possible that by leaving and rejoining the resistance frequently, they may end up protesting even more.

The Percent Immediate Protest parameter relates to the order of rules followed by an agent, as it determines how many civilians protest immediately. If a civilian protests immediately, they follow Rule NV, which is protesting, after Rule C, which is deciding to join the resistance. A civilian that does not protest immediately might become nonviolent on one turn, and then they will protest the subsequent turn. That said, they may be arrested before the second turn occurs or the situation in the agent’s neighborhood might change, such that they are no longer influenced to protest.

#### Rule D: Defection of pillars and policemen

A policeman or pillar defects to the nonviolent resistance when the following is true in their neighborhood:

$$\frac{number\;of\;protestors}{number\;of\;agents}\;>defection\;threshold$$

$$\frac{n_{pr}}{n_{a}}>dt$$

Each pillar has their own randomly assigned defection threshold, which is determined by the model parameters Defection Threshold as the mean value and Defection Threshold St Dev as the standard deviation. Most runs of the model have Defection Threshold St Dev set to zero. Once a pillar or policeman defects, they remain in defection.

#### Rule P: Policemen arrest or kill

This rule first checks if a policeman has defected. If so, they do not attempt to arrest or kill activists or nonviolent protestors.

Next, the policeman decides if he will target anyone. A random number is generated between 0 and 100, and if it is less than the parameter Chance Target Nonviolent, then the policeman will attempt to kill or arrest the nonviolent civilian or activist.

If there is a visible protestor, the policeman will target the protestor. If not, another random number is generated. If it is less than the parameter Chance Find Nonviolent, the policeman can target an activist or nonviolent civilian.

When targeting an individual, another random number is generated and compared to the parameter, Chance Kill Nonviolent. If the number is less than the threshold, the person is killed. If higher, they are arrested. Unlike Moro’s model [12], there is no policeman-precision parameter that determines the possibility of successfully killing. As the protesters are unarmed and in public, it is assumed the security forces always succeed in killing or arresting when they attempt to do so.

A crucial aspect of the model is that whenever a nonviolent civilian or activist is killed, then the government legitimacy variable slightly decreases. This is simulating backfire: a common process in which the public questions the government’s legitimacy in the aftermath of an atrocity—particularly when the police kill an unarmed activist [28–35]. It also aligns with the notion that governments that must resort to force to restore order demonstrate that they have less legitimacy than governments that compel the voluntary obedience of the population. Hence:

Newgovernmentlegitimacy=backfirecoefficient*governmentlegitimacy

$$GL_{new}=BC*GL_{current}$$

The backfire coefficient is equal to 0.99 in the models run for this paper, decreasing government legitimacy in the aftermath of a killing.

When an agent is arrested, they are assigned a jail time between 0 and the parameter, Max Jail Time. For these time steps, they cannot move, protest, or influence other agents to become nonviolent.

#### Rule M: Movement

All agents, except support pillars and those in jail, can move to an open spot in their visible neighborhood as determined by their vision parameter. This is unchanged from Epstein’s work [11], and it allows for changing neighborhoods.

As described in the Results section, we added a strategy component for the activists in which they purposefully move closer to pillars. If the model parameter named Pillar Prox Strategy equals 0, they randomly move like the other agents. If Pillar Prox Strategy equals 1, they move as close as possible to a regime pillar in their neighborhood. If Pillar Prox Strategy equals 2, they look for the pillar with the lowest individual defect threshold in an enlarged vision neighborhood which is defined by the parameter Activist Search Vision. They move as close as possible to that more vulnerable pillar, though they can only travel within their neighborhood each timestep.

#### Victory conditions

The model runs until a victory condition is achieved for the regime or the resistance, or until the model reaches a maximum allowable limit determined by the parameter Max Steps. The victory condition for the regime is the death of all activists, which assumes victory for the regime; the regime also wins if the model stops running before the resistance achieves its victory condition, meaning the status quo remains. Because nonviolent revolutions succeed by convincing key pillars of the regime’s support to defect, the victory condition for the resistance involves a certain percentage of pillars defecting. In reality, some defections are typically necessary but insufficient for movements to succeed [6, 7, 36]. Countries vary in terms of how many pillars are required to defect in order for a movement to succeed, and in which sectors the crucial pillars reside. For example, in Tunisia in 2010 there were defections among business and economic elites, labor organizations, professional groups such as attorneys and medical professionals, and senior security forces, which ultimately led to the ouster of President Ben Ali in 2011. In the case of Egypt in 2011, the key pillar that defected was the army, which refused to back Hosni Mubarak against the popular uprising that deposed him in February 2011.

To approximate this reality and match historical data, the percentage of pillars required to defect to meet the victory condition is one of the parameters we varied to match historical data; however, the minimum number of pillars required to defect for victory is 1%, and the maximum number is 80%. Recall that pillars (and police) defect when they have seen sufficiently large protests in their vicinity, contingent upon their individual defection thresholds based on their personal loyalty to the regime. We reiterate that we focus on defections because prior models that require all security forces to be killed for the movement to achieve victory cannot apply to models of nonviolent revolutions.

Having created the agent-based model, we calibrated model parameters to best match existing historical data. The data were matched against observations of all primarily nonviolent revolutions worldwide from 1945–2014. These data were drawn from the Nonviolent and Violent Campaigns and Outcomes (NAVCO) dataset and include all known instances in which there were at least a thousand observed participants mobilizing through protests, strikes, boycotts, and other unarmed methods to overthrow an incumbent national leader, constituting 110 cases in all [27]. For the purposes of this study, we exclude territorial resistance campaigns, such as anti-colonial and secessionist campaigns, which often have different social and spatial dynamics than anti-government campaigns, as well as campaigns that occurred alongside contemporaneous violent campaigns, as the presence of violence could alter the actions of police and pillars [9, 35]. Moreover, the data exclude social movements with reformist demands, such as the civil, political, social, or economic rights. As such, the scope of our study is limited to those campaigns with maximalist, or revolutionary, goals. The historical data include indicators on the peak participation of the campaign, whether security forces defected, and whether the campaign achieved the overthrow of the incumbent national leader within a year of peak mobilization [27].

We trained the model on the historical data to gauge model fit as we varied key parameters in the computational model. This allowed us to attune the parameters to the historical data. Once we completed this step, we also assessed the degree to which different strategies by activists and civilians improved model performance over historical outcomes. For details about this process, see the S1 Appendix.

## Results

Our expectation is that a resistance movement that starts relatively small and consists of a committed core group may increase its chances of success by expanding its numbers rapidly and consistently. But even when the movement remains small, we expect that the campaigns can improve their success rates relative to their participation size when they self-consciously target pillars of support by persuading them to defect or by disrupting their day-to-day lives. We also expect that their success in achieving substantial defections increases if they have access to information that a particular pillar’s loyalty to the regime is wavering.

The methods section shows the results of the training process, presenting the results from Model 1 also entitled "Initial Optimization." The parameters for Model 1 were optimized based on an evolutionary algorithm. To better fit historical data, the Defect Threshold and Nonviolent Success Percent were varied run to run, resulting in Model 2, entitled "Baseline." The following results are based on described variations to Model 2. Though every set of runs of the model produces slightly different results due to the stochastic nature of agent-based modeling, previous and subsequent sets of runs provide confidence that the presented case in the following plots are typical. Parameter values for all models are reported in S2 Table.

### Exploring a strategy to quickly mobilize recruits

**Fig 2:**
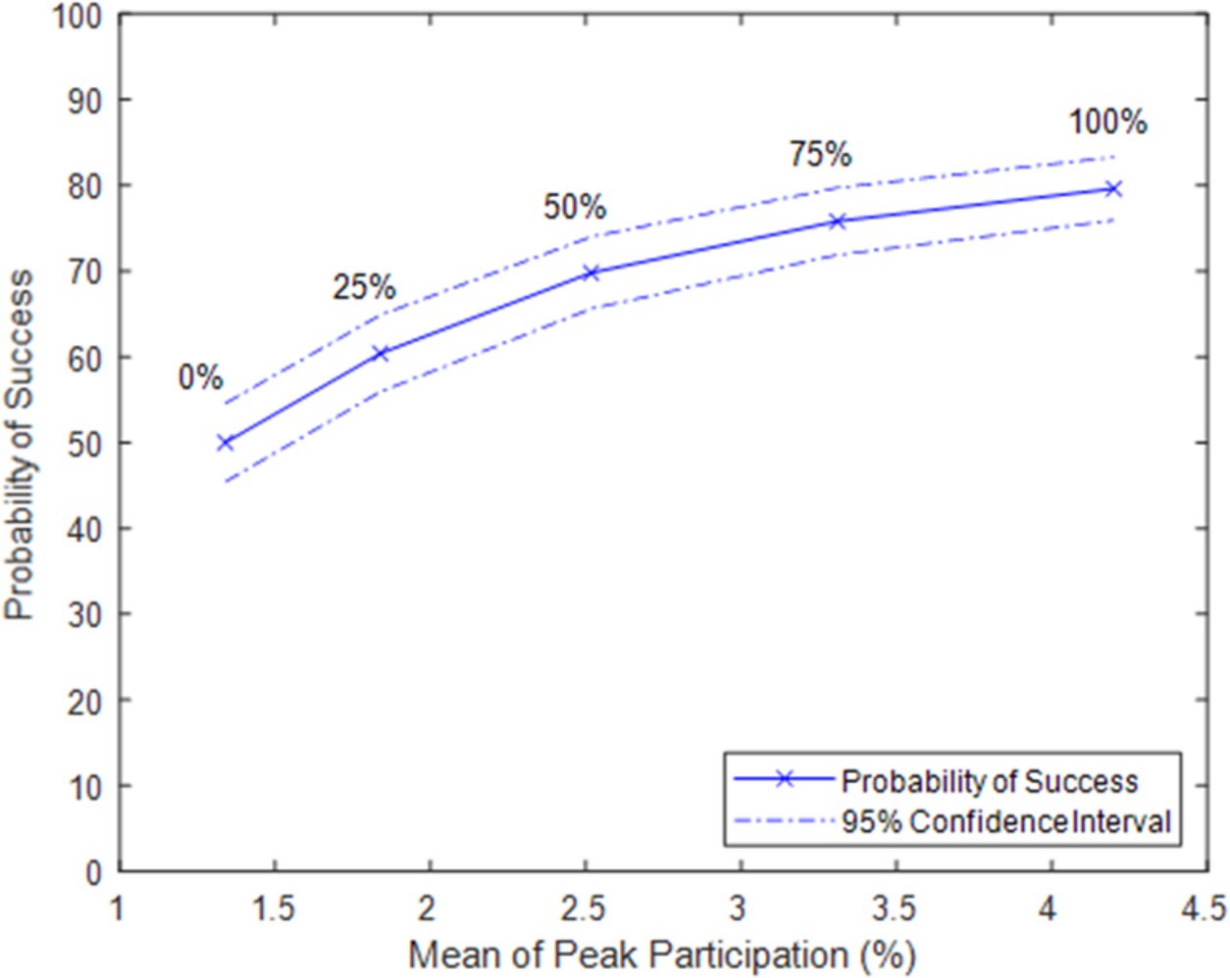
Participation and success increase with Percent Immediate Protest. Note: data points labeled with Percent Immediate Protest.

Having established a baseline model, we next explore potential resistance strategies by modifying variables, starting with the parameter Percent Immediate Protest. Increasing this value results in more civilians protesting immediately once they decide to join the nonviolent resistance. While this might reflect different protest proclivities among individuals, it could also flow from a strategy in which resistance leadership encourages immediate action from bystanders. Fig 2 shows that increasing the Percent Immediate Protest increases both the mean peak protest size as well as the probability of success:

When a turn takes place between joining the nonviolent resistance and protesting, it is possible that the agent might not protest, which in turn slows the momentum of other civilians joining and protesting. Hence, it follows that a higher Percent Immediate Protest would lead to larger protest sizes and increased success, as shown in Fig 2.

**Fig 3:**
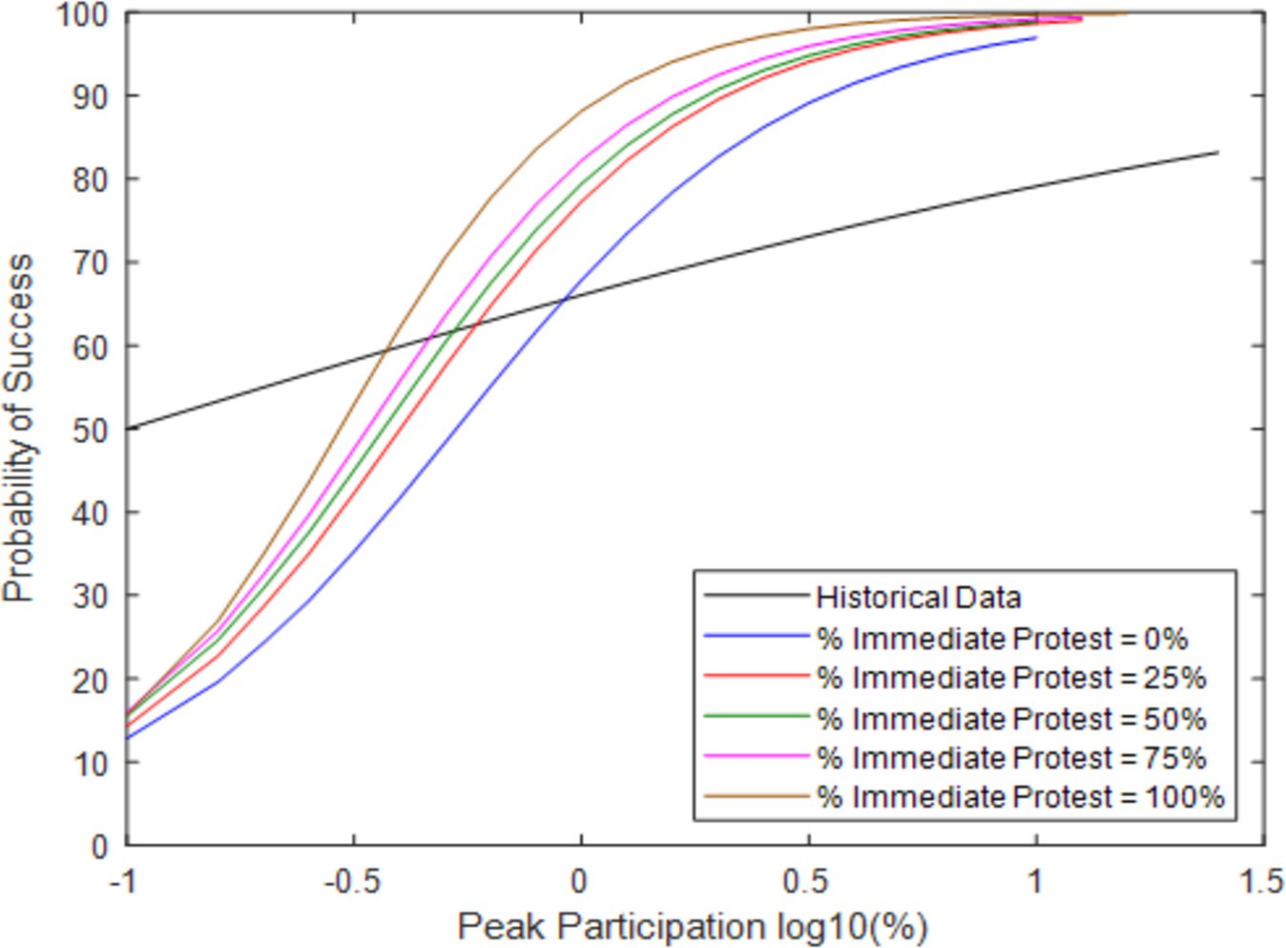
Probability of success at a given peak participation with varying Percent Immediate Protest.

While it clearly improves the overall likelihood of success, Percent Immediate Protest also affects the likelihood of success as a function of maximum protest size. (As discussed in the Appendix, the x-axis in Fig 3 and other figures shows the common logarithm—or logarithm with base 10—of the percent of peak participation, as opposed to the natural logarithm). For example, the likelihood of success is improved more when the peak participation is around 1% (log10 = 0) as compared to 0.1% (log10 = -1). This trend is intuitive as it also follows that this will only have its effect when the victory is dependent on a larger number of nonviolent civilians. When the peak participation is low, the outcome does not depend on whether a broad base of civilians mobilizes on behalf of the resistance. Either the activists succeed or not in causing pillar defection. When the peak participation is larger, then many civilians must have joined, so a parameter affecting the civilians can change the model outcome. This parameter increases the slope of the logistic regression; there are similar success levels for low protest size but more success for large protest size.

### Exploring a strategy of maintaining committed followers

As we swept the parameter Percent Immediate Protest, we also varied Percent Committed. When the parameter is set to 0, then none of the nonviolent civilians are committed, meaning that they cease to be part of the resistance when they do not see other agents protesting in their neighborhood. If they are not part of the resistance, they do not influence other civilians to protest.

**Fig 4:**
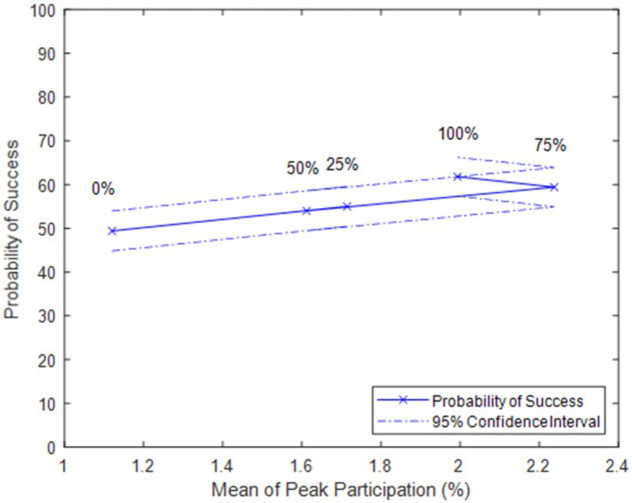
Participation and success mostly increase as percent committed increases. Note: data points labeled with Percent Committed.

**Fig 5:**
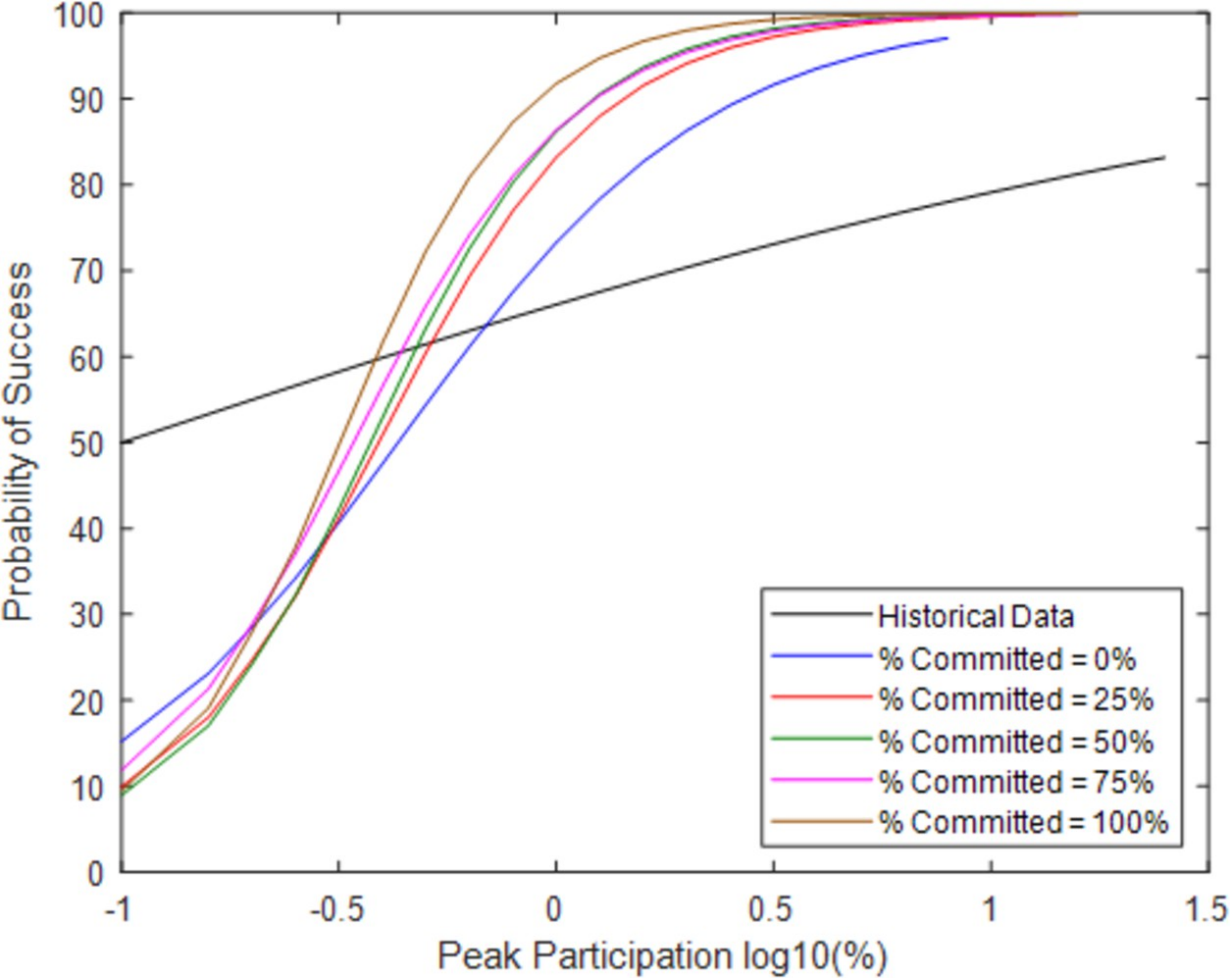
Probability of success at a given peak participation with varying percent committed.

While Percent Committed describes civilians’ level of commitment, it could be influenced by movements strategies. If resistance leaders kept their members active in influencing others even when they are not protesting, this would correspond to a higher value for Percent Committed. In Figs 4 and 5, we plot the effect of the parameter on peak participation, total number of successes, and probability of success for a given peak participation.

Though greater Percent Committed generally produces greater peak participation and probability of success, the increase is modest. This means that a greater Percent Committed might occasionally produce a lower mean peak participation, or a lower mean peak participation may correspond to a slightly greater probability of success. This is because the simulations depend on many randomly generated values. For example, though the probability of success for 100% Committed is higher despite the lower mean of peak participation, the 95% confidence intervals overlap. **A**gain, we see that the use of a strategy focused on maintaining the mobilizing influence of former protestors modestly increases participation and therefore the likelihood of success. But it does not increase the probability of success when few people join the resistance.

### Exploring two strategies focused on pillars

We have established that resistance strategies focused on recruits increase the likelihood of success in general, but not when the resistance is small. Consequently, we introduce two strategies focused on pillars in which activists focus on moving close to pillars in order to protest near pillars. Both strategies have two compounding effects. First, the activists themselves influence the pillars to defect. Second, the activists influence the civilians near the pillars to protest as well, which further influences the pillars.

**Fig 6:**
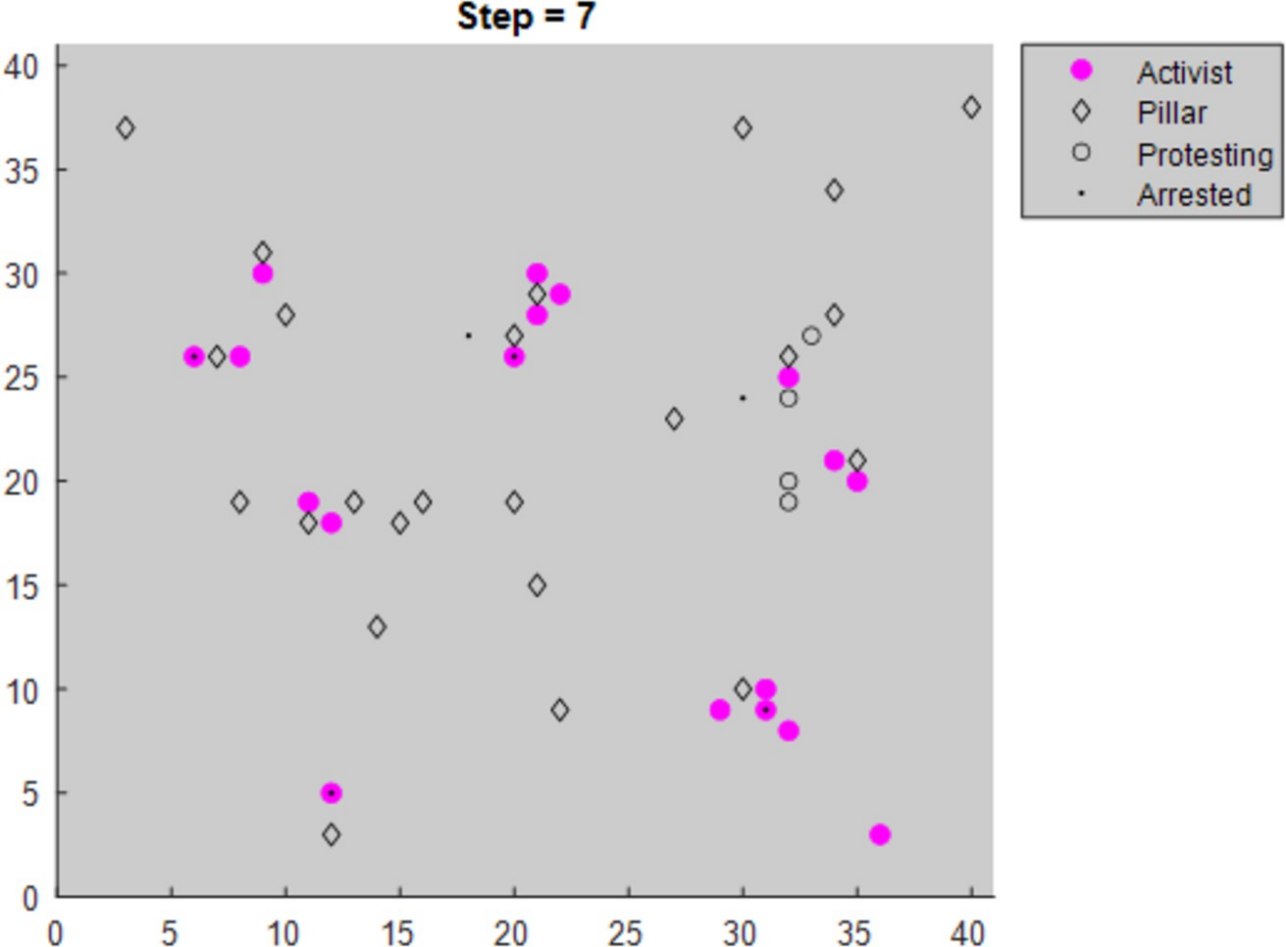
Activists using Pillar Prox strategy = 1 (Moving to the nearest pillar).

**Fig 7:**
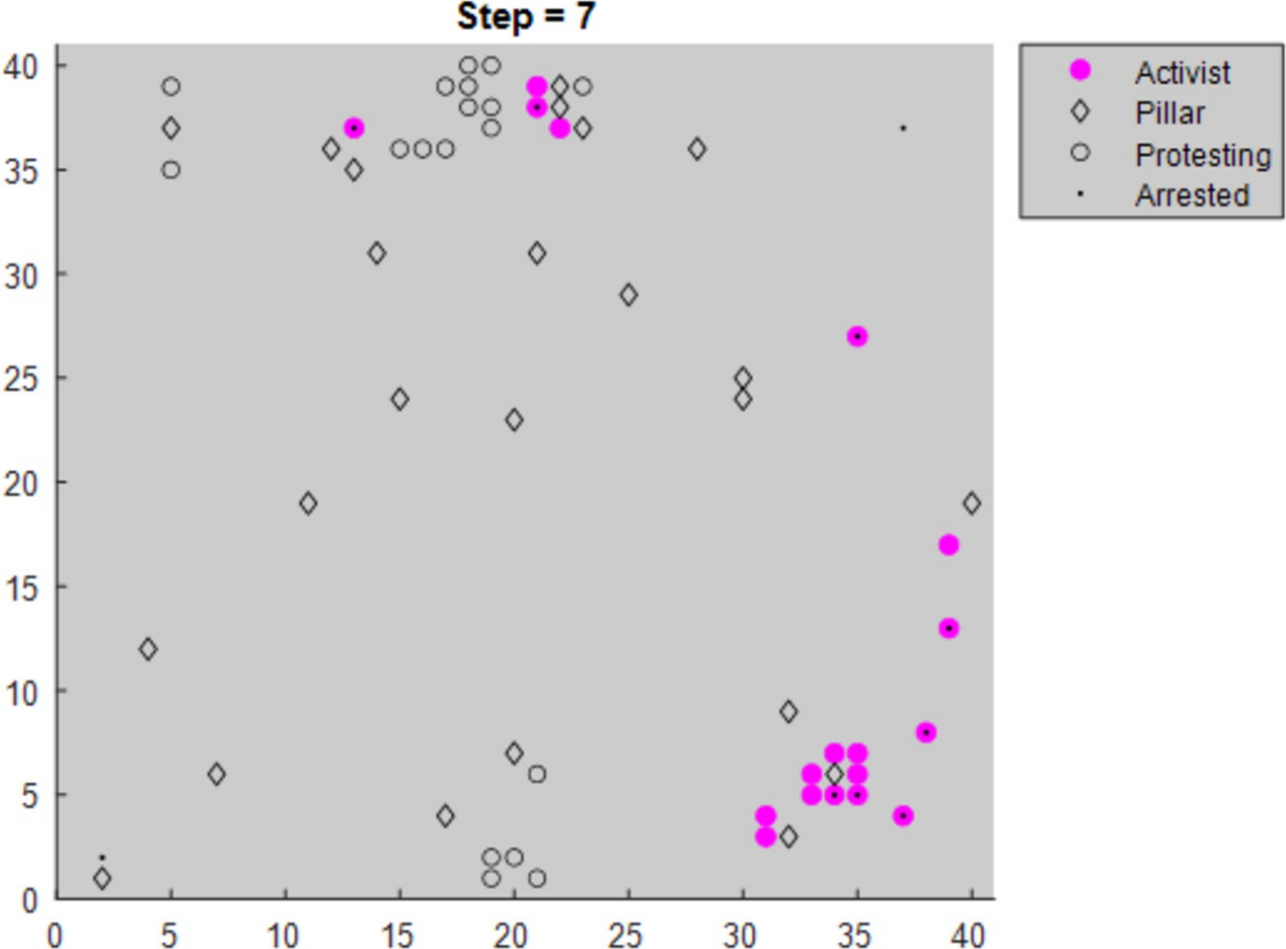
Activists using Pillar Prox strategy = 2 (Moving to the pillar with the lowest defect threshold).

These strategies are explained in the Methods section, but in short, they are as follows:

- When Pillar Proximity Strategy = 1, activists move to the nearest pillar (Fig 6).
- When Pillar Proximity Strategy = 2, activists move to the pillar with the lowest defect threshold in their expanded vision region (Fig 7).

Modified graphs display the activist and pillar agents for clarity.

**Fig 8:**
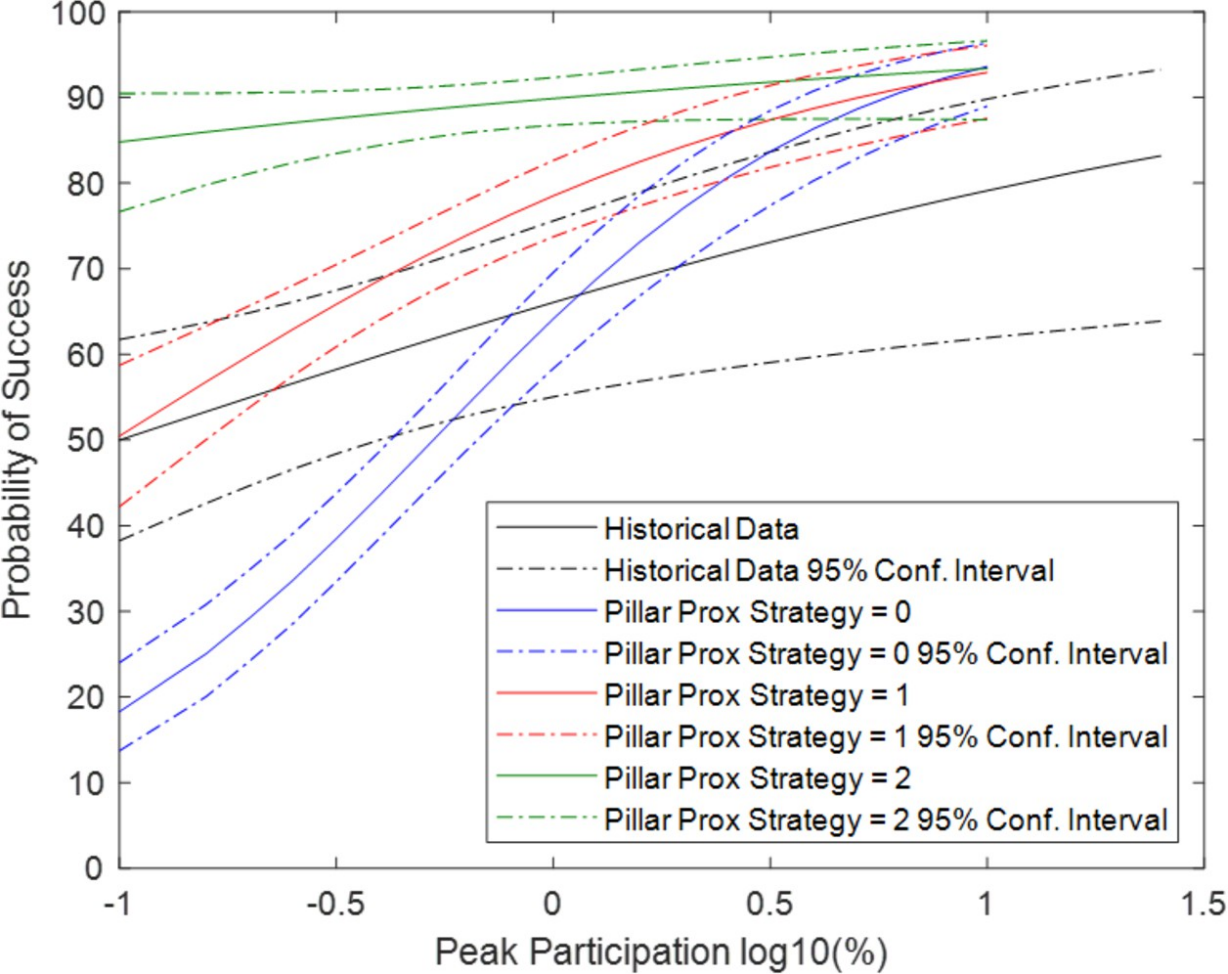
Comparing probability of success for Pillar Proximity Strategies.

Fig 8 compares the probability of success for Pillar Proximity Strategies 1 and 2 against activists moving randomly, without a strategy with regard to pillars (Pillar Proximity Strategy = 0). As Pillar Proximity Strategy 2 requires individual pillars to have varying defect thresholds, Fig 8 includes varied individual defect thresholds as defined by the parameter named Defect Threshold Std Dev.

The average success probability is 48% for no strategy, and 68% and 88% for strategies 1 and 2, respectively. In other words, these pillar proximity strategies clearly increase the probability of success. But the most notable implication is that they also increase the probability of success when the peak participation is very low. Indeed, the most significant improvement in the probability of success is seen when the peak participation is only 0.1% (log10 = -1). Under these conditions, the probability increases from approximately 20% to 50% and 85% for strategies 1 and 2, respectively. In other words, even when a small, committed group of activists are unable to recruit large portions of the populace to their cause, they greatly increase their probability of success by focusing on the pillars of regime support. Furthermore, when the peak participation is sufficiently large, then the pillar strategies have little to no effect. People are protesting in enough places such that focusing on pillar locations is not needed.

## Discussion

Our study calibrated a dynamic agent-based model to historical data, which allowed us to develop a model that could assess the impacts of movement strategies on movement outcomes. We explored three strategies. The first focused on rapid mobilization, and the second focused on maintaining recruitment activity of nonviolent civilians. Both increased peak participation and movement success for larger campaigns, but these strategies do not increase the probability of success for movements that remain small. But the third strategy—which activates protest in proximity to key pillars and, alternatively, can deploy intelligence about which pillars are the most likely to defect—makes even smaller campaigns more likely to succeed than campaigns that grew very large in size without implementing such a strategy. This finding allows us to explain how and why some smaller resistance movements are able to succeed by deliberately incorporating efforts to dislocate the regime from its pillars of support, even where larger movements without such strategies fail.

There are a few theoretical and practical limitations to the current study. First, the scope of the study is limited to anti-government campaigns in which we assume an equal degree of social distance between agents throughout the model, and in which protestors face active government repression. Current research suggests that key pillars are less likely to defect—and movements are less likely to succeed—when there is a high degree of social distance between the resistance and the regime [37]. This could emanate from, for example, racial, ethnic, class, caste, and/or gender differences between protesters and police, and may be particularly pointed in highly unequal or hierarchical settings. Less repressive regimes, or regimes who repress unevenly across groups, may not face the same backfire effect captured in the model. Moreover, some regimes may actively try to prevent defections from different pillars, using various social or financial inducements; such strategies may help to explain variations in the model’s fitness to the historical data. Future extensions of the model could attempt to account for these strategies as well.

Second, the study assumes that government legitimacy decreases with police killings. There may be important caveats to this assumption. For instance, government legitimacy may only decrease among those most proximate to it (e.g. within the neighborhood of the killing), or among those with shared identities as the victim. Alternatively, the population could react to police killings in a polarized way, with police killings increasing perceived legitimacy among some segments of the population (e.g. regime loyalists or those belonging to a different social identity group than the protesters), while decreasing it in others [38]. Studies that extend this model could explore these potential variations by altering model parameters regarding government legitimacy.

Third, the only resistance tactic available to the agents in the model is protest, though it is likely that different tactics would have varied effects on pillars. For example, an economic or business pillar might be more persuaded by a boycott action than a protest. In this way, the neighborhood of the pillar does not represent a geographic region but symbolizes the sphere of society that matters to them. Further research could assess the effects of different resistance tactics on various pillars using more fine-grained historical data.

Fourth, further research could explore how pillars interact with one another, as well as which pillars might be more influential in creating defection cascades. For instance, instead of basing the nonviolent victory condition on the number of pillars defecting, an agent-based model could instead weight each pillar with a different number of points. This would approximate the reality that there are key sources of social and political power in society—for instance, business elites and security forces have an unequal impact on the outcome depending on the country and regime type. Adding this complexity means that the model would not need to vary victory conditions from run to run, as it could instead vary pillar attributes from run to run.

Our study nevertheless provides important new information about the efficacy of mass mobilization and strategic nonviolent action. Because previous literature has emphasized that the likelihood of success largely depends on the size of the resistance movement, activists might focus all their effort on growing their membership and increasing participation in nonviolent action. But our agent-based model points to key factors about the regime that significantly influence movement success. Specifically, it matters how close to defection the regime’s support system is. If a few pillars are close to defection and could easily sway the others, then the activists can achieve success even at small participation sizes. If the pillars are very committed to the regime, then the activists have a much harder task ahead of them. Here, the model shows that motivating people to protest immediately may be as important as garnering sustained commitment.

At the same time, the model results point to a way that the activists can exercise their own agency, even when the regimes support pillars are not initially inclined towards defection. Our results encourage resistance leaders to evaluate who the most important pillars of society are; how likely they are to defect; and whether they have divided loyalties. The more information the resistance has on the latter, the more likely they can apply the lessons from Pillar Proximity Strategy 2. While Pillar Proximity Strategy 2 (i.e. focus efforts on pillars most likely to defect) yields the greatest success, it is also unrealistic without credible intelligence about the pillar’s loyalties.

These findings therefore point to two crucial capacities for successful movements in repressive regimes. First, the ability to organize and coordinate a viable resistance strategy—particularly one that is oriented toward eliciting defections—may help movements to succeed. Second, the ability to gather credible information about particular pillars’ propensity to defect may allow the movement to use the most effective strategy available, which is coordinated resistance toward wavering pillars. More generally, movements with more centralized informational structures may be more likely to succeed than leaderless movements, which may excel at mobilizing mass participation but fall short when it comes to coordinating a clear strategy and processing intelligence. In some instances, the general public might have significant amounts of information regarding the pillars, their likelihood of defection, what could influence them, and which are the most influential. These are often called “leaderful” movements. If transnational activists and solidarity networks want to support nonviolent campaigns, research and information-gathering on the political and economic values of key pillars could be a fruitful avenue of support.

## Supporting information

S1 Appendix Appendix. (DOCX)

S1 Fig Example line graph. (TIF)

S2 Fig Probability of success for given participation size. (TIF)

S3 Fig Raw data of only nonviolent resistance, regime change. (TIF)

S4 Fig Historical data of peak participation sizes. (TIF)

S5 Fig Historical data of peak participation sizes, logged. (TIF)

S6 Fig Probability of success for given peak participation for historical data. (TIF)

S7 Fig Effect of R2R_DefectThresholdMin on error terms. (TIF)

S8 Fig Probability of success for given participation size for three runs of baseline case. Error = 9.48, 13.0, and 8.7 respectively.
(TIF)

S9 Fig Raw data of optimally matching case including strategy. (TIF)

S10 Fig Histogram of peak participation size of optimally matching case including strategy. Error = 0.62.
(TIF)

S11 Fig Probability of success for given participation size of optimally matching case including strategy. Error = 2.21.
(TIF)

S12 Fig Comparison of methods to match historical data. (TIF)

S13 Fig Raw data of initial optimization model. (TIF)

S14 Fig Histogram of peak participation size of initial optimization model. Error = 0.33.
(TIF)

S15 Fig Probability of success for given participation size of initial optimization model. Error = 15.3.
(TIF)

S16 Fig Example distributions of Defect Threshold and Nonviolent Success Percent. (TIF)

S17 Fig Raw data of baseline model. (TIF)

S18 Fig Histogram of peak participation size of baseline model. Error = 0.50.
(TIF)

S19 Fig Probability of success for given participation size of baseline model. Error = 9.37.
(TIF)

S20 Fig Overall probability of success for each defect threshold value. (TIF)

S21 Fig Probability of success at a given peak participation. (TIF)

S22 Fig Overall probability of success for each defect threshold value. (TIF)

S23 Fig Probability of success at a given peak participation. (TIF)

S24 Fig Probability of success for given participation size with 50% of runs using Pillar Prox strategy = 1. (TIF)

S25 Fig Successes and failures with and without strategy (Pillar Prox = 1). (TIF)

S1 Table Model parameters. *Note that density is not percentage of the populace, but percentage of allowable spaces in the torus lattice. **The MATLAB model uses a variable entitled Percent Fickle, whose value equals 100—Percent Committed. For the sake of clarity, this article uses the term Percent Committed.
(DOCX)

S2 Table Parameters and results for experiments 1–6. (DOCX)

S3 Table Parameters and results for models 1–4 & experiments 7–8. (DOCX)

S4 Table File summary. *Files can be easily modified to run with or without MATLAB Parallel Computing Toolbox, which decreases computation time when used. **Uses MATLAB Statistics and Machine Learning Toolbox.
(DOCX)
