# Supplementary material for: A dynamic model of nonviolent resistance strategy
Source: PLoS One. 2022 Jul 27;17(7):e0269976. doi: 10.1371/journal.pone.0269976 (PMC9328538; doi:10.1371/journal.pone.0269976)
Supplement: S1 Appendix — (DOCX) [file pone.0269976.s001.docx]

**Appendix**

The initial version of the model was created in NetLogo, but later was moved from NetLogo to MATLAB code for greater flexibility. The agent-based model is simulated when the MATLAB m-file is run named ResistanceABMFinal.m. This can be run directly from MATLAB’s command window or called by another MATLAB function.

As inputs, ResistanceABMFinal.m takes in model parameters, which are described below, or it can run with default parameters. It also outputs information including victory condition, number of time steps run, peak protest size, etc. It can also generate plots including the model graph (i.e. grid with agents on it) at a given time step (Figure 1) and line graphs showing the total number of various agents over time (Figure S1).

ResistanceABMFinal outputs the victory condition and distinguishes between a resistance loss or timing out. When determining the probability of success graph, however, timing out is counted as a loss. Clearly, based on Figure S2, we have more consistent results when we regard timing out as a resistance loss. As almost all successes occur prior to the 200th time step, Max Steps is typically set to 200.

**Effects of Rule Order and Other Notable Model Functionality**

All agents are created prior to the initial time step and given their appropriate settings such as agent type, income, hardship, etc. Their initial placement is considered time step 1, and the earliest any agents can act is time step 2.

Early testing of this model found that almost all of the activists would immediately protest in the timestep 2 or 3, creating a peak participation that often did not occur again in the simulation if the resistance failed. This falsely increased the peak participation value for many of the failed runs. Consequently, a parameter called Delay Start Max was added. Each agent is delayed a certain number of time steps randomly assigned between 1 and Delay Start Max before they can act in any way.

When an agent can follow multiple rules in a time step, the order of the rules can have a significant effect. All agents that can move will first move to an open space in their visible neighborhood (per Rule M). Some civilian agents first check to see if others will join or remain part of the resistance (per Rule C), and then if so, decide if they will protest (per Rule NV). Other civilians, if they are nonviolent from a previous time step, will first decide whether or not to protest (per Rule NV) before deciding whether or not stay part of the nonviolent resistance (per Rule C). The model parameter Percent Immediate Protest determines what percent of the civilians do Rule C then NV, or in the other order. Lastly, police can arrest or kill (per Rule P) before they decide to defect (per Rule D).

For each time step of the model, each agent has the opportunity to perform its corresponding rules once. Originally, the order of action is all civilians, all activists, all police, and finally all pillars. If the Reorder Agents Parameter is set to 1 in order to avoid any undesired systematic effect of this order, the first three agent types are reordered at the start of the model, but the pillars still go last. Pillars do not affect the other agents.

Consequently, it is inevitable that some protesters will be killed or arrested before the pillar has an opportunity to see them (and also before the user of the code can see them on the graph as well). Nevertheless, to have an accurate count of all protestors in a time step, a counter is used which is incremented for each agent that decides to protest or continues to protest in that time step, even if they are later killed or arrested. This produces a slightly larger peak protest size than if the number of protesters were simply summed at the end of the time step.

**Model Parameters**

Table S1 contains the parameters used in simulations along with typical values, a description, and the rationale for each value. The typical values were either maintained from Moro [12], were considered reasonable in themselves, or selected to produce realistic outputs. The one exception to this is “Percent Fill Police,” which we selected based on historical data of nations’ police and military sizes. But even this was increased from a mean value of approximately 2% to 4% on the assumption that repressive regimes would have larger security forces; moreover, this matched Moro’s value of 4%, which further validated our choice [12]. In some cases, the parameters are varied in each run of the model. The actual values used in each case presented in the Results section can be found in Table S2.

**Statistical Analyses**

The first step in matching the historical data is creating the histogram for peak participation size and the model of the probability of success for a given peak participation size, which is done in the file GetNAVCOData.m, drawing from the NAVCO data [27]. Counting all partial successes as losses, Figure S3 shows successes and losses for the nonviolent resistance. Figure S4 plots histograms for peak participation percentage and the log of peak participation. To interpret the second subplot in Figures S3 and Figure S5, -1 corresponds to 0.1%, 0 corresponds to 1%, and 1 corresponds to 10%. The common logarithm or base 10 logarithm is used instead of the natural logarithm for ease of interpreting the graph.

To calculate probability of success, we used a logistic regression on the historical data. Instead of determining the probability of success as a function of peak participation, we instead determined it as a function of the log of peak participation. This puts greater focus on the population sizes that are more common.

This process leads to the historical model the agent-based model attempts to match (Figure S6). Due to the size of the agent-based model lattice, it is not possible to have protest sizes smaller than .08%. Consequently, data of probability with the log of peak participation less than -1 is ignored.

**Optimizing Model Parameters and Determining Fitness**

To create simulation output, ResistanceABMFinal.m is run between 200 to 500 times, and there are two outputs. First a normalized histogram is created that counts the number of runs for a given range of peak participation sizes divided by the total number of runs which had a protest. Any runs in which no activists ever protested are discounted. Second, the logistic regression is created as a function of the log of the peak participation.

Each run could be considered a different instance of resistance in various fictional nations and times. In order to have varied population sizes equivalent to the historical data, the Percent Fill Activists parameter is varied each run. All cases in this report use a mean Percent Activist Fill of 0.8% with a standard deviation of 0.3%.

As mentioned in the results, other model parameters are occasionally varied from run to run. These include Defect Threshold, Nonviolent Success Percent, and Pillar Proximity Strategy. Defect Threshold, and Nonviolent Success Percent are determined based on a chi-squared distribution utilizing new parameters. For example, the Defect Threshold for each run is determined using the equation:

$$Defect Threshold = R2R\_DefectThresholdMin + R2R\_DefectThresholdSTD*{(RandomNumber)}^{2}$$

The random number is generated by MATLAB based on a normal distribution with mean = 0 and standard deviation = 1. If the Defect Threshold exceeds a value determined by R2R_DefectThresholdMax, then it is set to the maximum value. The same is done for Nonviolent Success Percent with similar parameter names.

When Pillar Proximity Strategy varies each run, then a random number is generated between 0 and 100 for each run, and compared to a parameter named Pillar Prox Strategy Ratio. If the random number is less than the parameter, the run uses Pillar Prox Strategy = 1.

To calculate the fitness of the model, two error values are calculated, one based on the peak participation histogram and one based on the probability of success logistic regression. In each case, the error is the summation of the square of the difference between agent-based model and historic data, which is then normalized by the variation in the historic data (i.e. the sum of the squared difference between individual historical data points and the historical mean). In other words, it is the term subtracted from 1 in an R^2^ value. Consequently, the error needs to be minimized.

There are multiple ways to fit the data. The first sweeps through multiple parameters and simulates all possible cases. The second uses randomly created cases and an evolutionary algorithm.

The sweep method is accomplished with SweepMethodFinal.m. The user enters all the values for all parameters, depending on the number of values they want to simulate. For example, if the user specifies to vary Defect Threshold as 0.05, 0.1, and 0.15 and vary Nonviolent Success Percent as 25 and 50, while providing single values for all other parameters, the code would simulate 6 cases:

- Defect Threshold = 0.05, Nonviolent Success Percent = 25%
- Defect Threshold = 0.10, Nonviolent Success Percent = 25%
- Defect Threshold = 0.15, Nonviolent Success Percent = 25%
- Defect Threshold = 0.05, Nonviolent Success Percent = 50%
- Defect Threshold = 0.10, Nonviolent Success Percent = 50%
- Defect Threshold = 0.15, Nonviolent Success Percent = 50%

If one wanted to vary 10 parameters with 5 values, that would be 5^10^ cases. Hence, the sweep approach is very good for exploring the effect of each variable, but not useful for determining optimal parameters.

The evolutionary algorithm is accomplished with EvoMethodFinal.m. As before, the user enters their best guess for each model parameter. For the parameters they choose to vary, however, they also enter a standard deviation to create random cases and a minimum allowable value. For instance, if the user enters the Defect Threshold as [0.05 0.02 0.03], the code will generate multiple values for the Defect Threshold in which the mean is 0.05 and the standard deviation is 0.02. If any values are less than 0.03, then these become 0.03. (Consequently, the true mean will not be 0.05, though it remains the peak of the truncated normal curve.)

EvoMethodFinal.m creates 20 randomly generated cases. As in SweepMethodFinal.m, each case is run multiple times and they are compared to the historical data. After this, 19 new cases are created using the previously best case as the new mean values and sometimes a reduced standard deviation. These 19 new cases and the previous best case are then simulated. This continues until a specified maximum number of iterations or the agent-based model’s fit of the data is deemed close enough.

As there are two error terms, one corresponding to the histogram of normalized participation sizes and one corresponding to the logistic regression of success probability, picking the optimal design is nontrivial. In most cases, the code looks for the smallest error for the histogram until one or more designs meet an error threshold, which is usually 0.8. Of these cases, it picks the case with the lowest logistic regression error. In other words, it makes sure the model’s participation sizes are close enough to the historic data, and then optimizes for probability of success for a given participation size.

Due to the two error terms, choosing the optimal matching is a subjective process. Below, we plot both error terms as a function of varying R2R_DefectThresholdMin in Experiment 5. This parameter determines the lowest possible value in the Defect Threshold chi-squared distribution. As Figure S7 shows, we could choose another parameter value that reduces the probability of success error, but it results in a worse peak participation size error. To explain why, decreasing the lowest Defect Threshold value results in more success when protest sizes are small, better matching the historic data logistic regression. However, this results in more runs with lower peak protest sizes, which differs from the historical data histogram.

With ideal computing resources and time, the evolutionary algorithm would be sufficient for finding the optimal matching. Ideally, hundreds of cases would be run each iteration, and each case would be run 500 to 1000 times. As this is unfeasible given current resources, the actual matching process makes use of both the EvoMethodFinal.m and SweepMethodFinal.m. Once a user gets close to the optimal parameter values with the evolutionary algorithm and has an idea which values have the largest effect, they can finetune by modifying values using the sweep method. The advantage, of course, is that this manual process forces the user to begin to understand the effect of the parameters.

As previously mentioned, creating repeatable logistic regressions and error values requires approximately 500 runs. The baseline case (Model 2) is run three times, and the curves of the logistic regression are close to one another, mostly inside their 95% confidence intervals. As the results differ slightly, they are recorded as Experiment 6 in Figure S8. Though more repeatable results could be determined from 1000 runs, that would increase the calculation time from approximately 1.5 hours per case to 3 hours per case with the current computer and hardware. As with any other computational modeling, there is a trade-off between precision and efficiency.

**Final Matching to Historical Data**

While the Results section referenced a baseline model without using the Pillar Proximity Strategy, we wanted to highlight the best possible match found, which we show with Model 4. By varying Pillar Proximity Strategy each run, we can create an improved baseline model. Using the previous baseline model and sweeping a new variable called Pillar Prox Strategy Ratio, five cases are created in which 0%, 25%, 50%, 75%, or 100% of the runs use Pillar Prox Strategy = 1. The 75% case gives the best matching (Figure S9).

Figure S10 shows the number of cases for a given protest size divided by the total number of cases in the chart. Each histogram bin includes the cases that are +/- .25 the number shown. For example, a number of cases in the bin at X = 0 includes the sum of cases between log10 of Peak Participation Percent between -.25 and +.25. This corresponds to all cases with peak participation between .56% and 1.78%. Again, this sum is divided by the total number of cases.

As the model cannot produce protest sizes smaller than one agent on the 40x40 lattice, the smallest protest size is approximately 0.08% which corresponds to logged value of -1.1 (Figure S11). Consequently, the historical data with smaller population sizes are not included in the normalization of the historical data.

In summary, we can see the best matching scenarios, namely Models 1, 2, and 4 as listed in Figure S12. While it may be possible to improve the model to better match historical results, adding the variation between runs creates a more realistic model. This found that the most important parameters to vary include those related to pillar defection, specifically when each pillar decides to defect, how many defections are needed for the regime to fall, and a strategy related to defection.

In summary, while many parameters can be changed to yield similar results with regard to overall probability of success and mean participation sizes, these parameters were not critical to producing results similar to the historical probability of success as a function of peak participation size. Instead, the critical parameters mentioned above were optimized to match all the key outcomes.

**Initial Attempt to Match Historical Data**

From multiple runs of the Initial Optimization model, we plotted the raw data of outcome as a function of peak participation size (Figure S13), histogram of peak protest sizes compared to the NAVCO 1.2 data (Figure S14), and probability of resistance success as a function of the peak participation size compared to NAVCO 1.2 data (Figure S15). Clearly, this model has more cases with smaller peak protests than the historical data. Though it was possible to generate model data with larger peak protests, this hurt the probability of success results, and this model was selected as the best matching.

Fig S15 shows the probability of success for a given peak participation size using a logistic regression. It is evident the model produced a much steeper curve for the probability of resistance success. In other words, it is difficult for the model to produce successes when the resistance had small protests and difficult to produce failures when the resistance was large. One possible explanation for this is that each run of the model only varied the starting activist size, but if each run represents a different fictional country and a fictional time, then we should expect many of the parameters to vary from run to run. Consequently, we began to vary some parameters, such as Starting Government Legitimacy and Defect Threshold during each run.

Certain parameters improved the fit, while others did not. For example, Starting Government Legitimacy would change the size of the peak participation, but not the likelihood of success for a given peak participation size. In other words, some parameters affect how many people join and others affect how the pillars are influenced for given protest sizes.

Consequently, we began to vary the Defect Threshold and Nonviolent Success Percent parameters for each run. Instead of varying the mentioned parameters with a normal distribution, we used a chi-squared distribution with one degree of freedom. This allows many low values to allow for successes when there is little peak participation, but still creates cases that make success for the resistance difficult when there is large peak participation. Outliers, however, cause a problem. If either value is too large, success becomes impossible. This results in cases with very large protest populations that still fail. Consequently, Defect Threshold and Nonviolent Success Percent are limited to maximum values of .3 and 80% respectively. Figure S16 plots the distributions for both parameters.

By varying Defect Threshold and Nonviolent Success Percent for each run as described, the probability of success plot matches historical data more closely. Figures S17, S18, and S19 show the newly optimized matching results for Model 2, also known as the Baseline Model. While more optimal parameter values might exist, this fit shows considerable improvement over the case in which all parameters remain the same each run. Looking at the raw data, we easily see that the model now produces a considerable number of failures for protest sizes larger than 1%.

**Further Understanding for Defect Threshold and Nonviolent Success Percent**

To understand the effect of the Defect Threshold and Nonviolent Success Percent parameters, we plot them against the probability of success.

First, we show a varying Defect Threshold while using the chi-squared distribution for Nonviolent Success Percent as Experiment 7. In other words, each datapoint in Figure S20 corresponds to a different case or set of 500 runs. In each case, every run uses the Defect Threshold pertaining to the value on the X-axis. Nonviolent Success Percent, on the other hand, varies run to run as it does for the baseline model.

Success can be achieved when 100% of the pillars must defect. In other words, even if all of the pillars must be swayed by the populace and all are needed to reject the regime, success is still possible, albeit unlikely. Defect Threshold creates a larger problem for the resistance. If the pillars require a majority of the people in their proximity to be protesting (i.e. the Defect Threshold > 0.5), then the resistance inevitably fails (Figures S20 and S21).

Next, we vary Nonviolent Success Percent, while using the baseline chi-squared distribution for Defect Threshold, as Model 4 (Figure S22). To clarify, the resistance succeeds when the number of pillars that defected exceed the Nonviolent Success Percent. Hence, the rightmost value in the plot is 99% (Figure 23). Although it is not plotted, if the parameter is set to 100%, then there is no possibility of success.

**Further Understanding for Pillar Proximity Strategy**

In the main text, all runs of the model used the same pillar proximity strategy, but this can also be varied run to run. Figure S24 shows results for Model 3, in which approximately 50% of the runs include Pillar Proximity Strategy 1 and the others have strategy 0. These results are summarized in Figure S25. Each subplot contains one third of the cases, with the top subplot containing the runs with the smallest peak participations and the bottom subplot containing the runs with the largest peak participations. The Y axes show the percent of runs that either succeeded or failed for a given strategy. In other words, the bar showing the percent of “Success With Strategy” is the number of successes with the strategy divided by the number runs with the strategy, not divided by the total number of runs. The red lines show the 95% confidence interval. Overall, most successes among low or mid-sized resistance movements required the strategy, while it has less of an impact for larger protests.

#### **Data Availability**

The Excel dataset and custom code that support the findings of this study are publicly available from the Harvard Dataverse at: <https://dataverse.harvard.edu/dataset.xhtml?persistentId=doi:10.7910/DVN/ME8JJZ>.
