# Supplementary figures and images for: A dynamic model of nonviolent resistance strategy
Source: PLoS One. 2022 Jul 27;17(7):e0269976. doi: 10.1371/journal.pone.0269976 (PMC9328538; doi:10.1371/journal.pone.0269976)

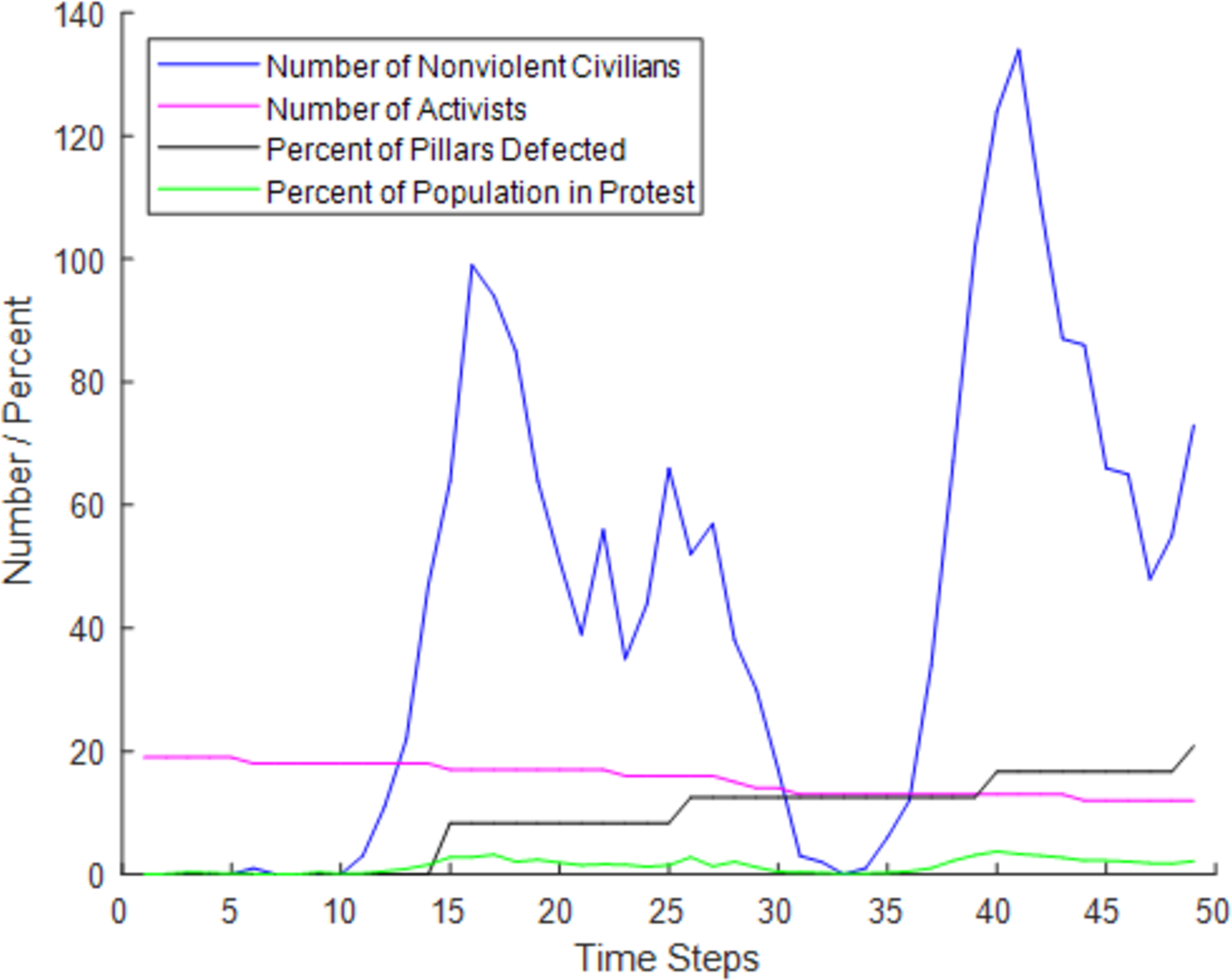

Supplement: S1 Fig — (TIF) [file pone.0269976.s002.tif]

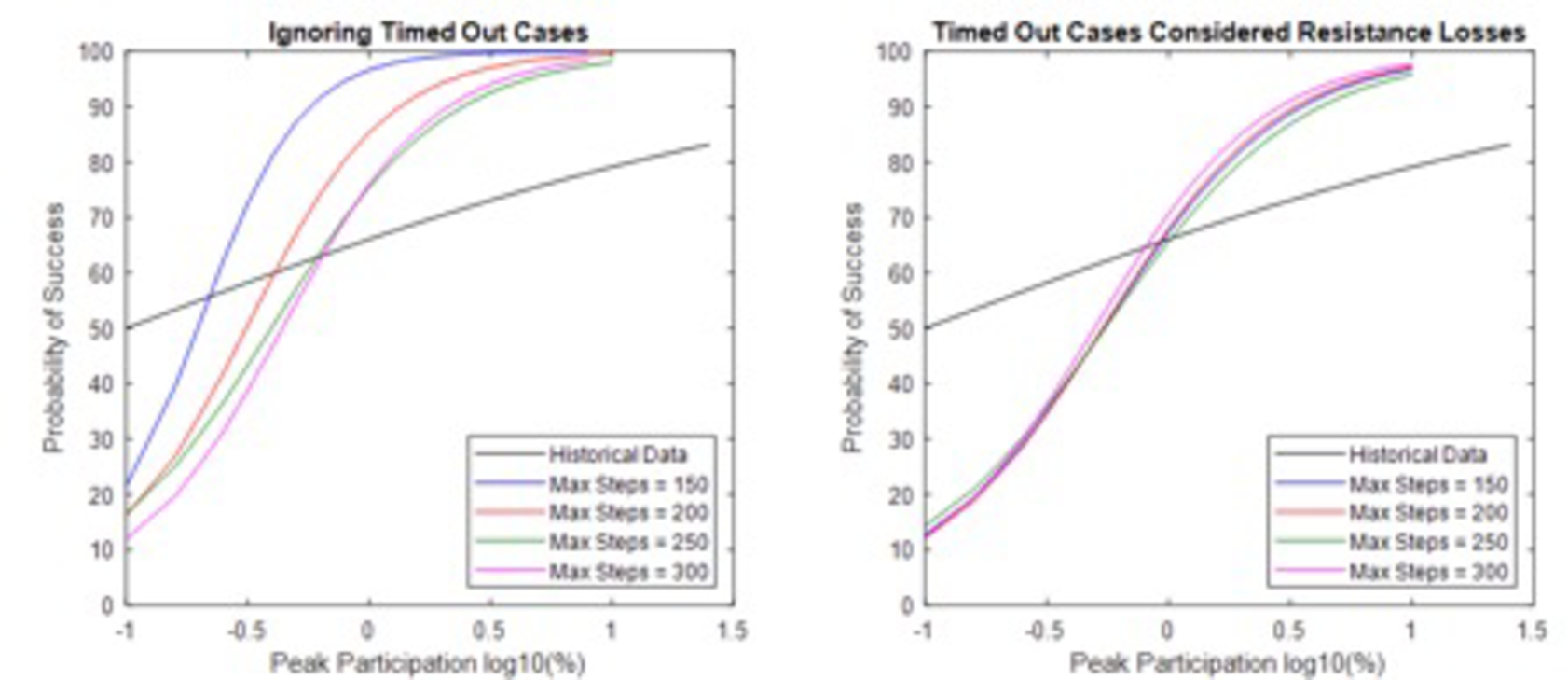

Supplement: S2 Fig — (TIF) [file pone.0269976.s003.tif]

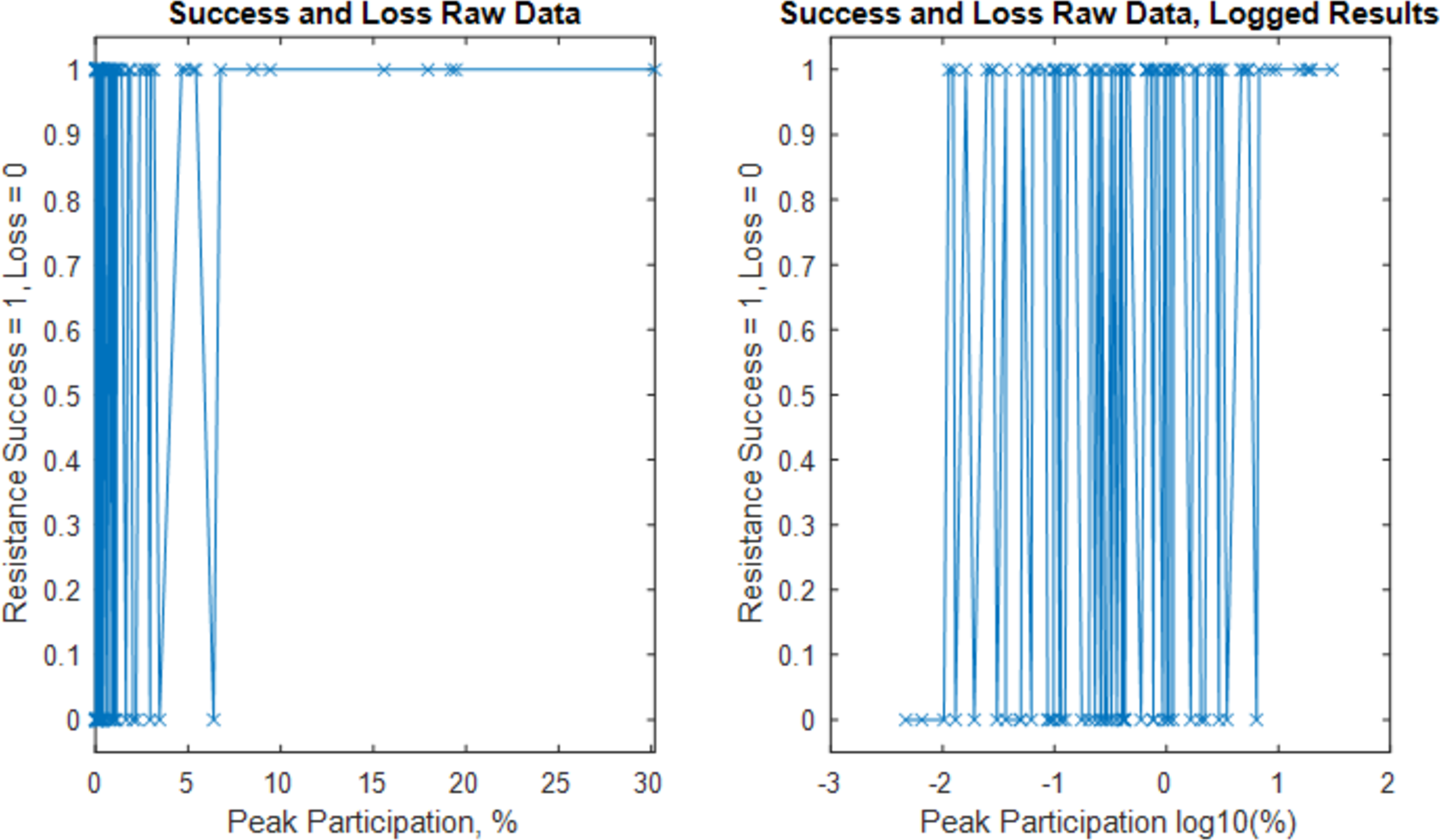

Supplement: S3 Fig — (TIF) [file pone.0269976.s004.tif]

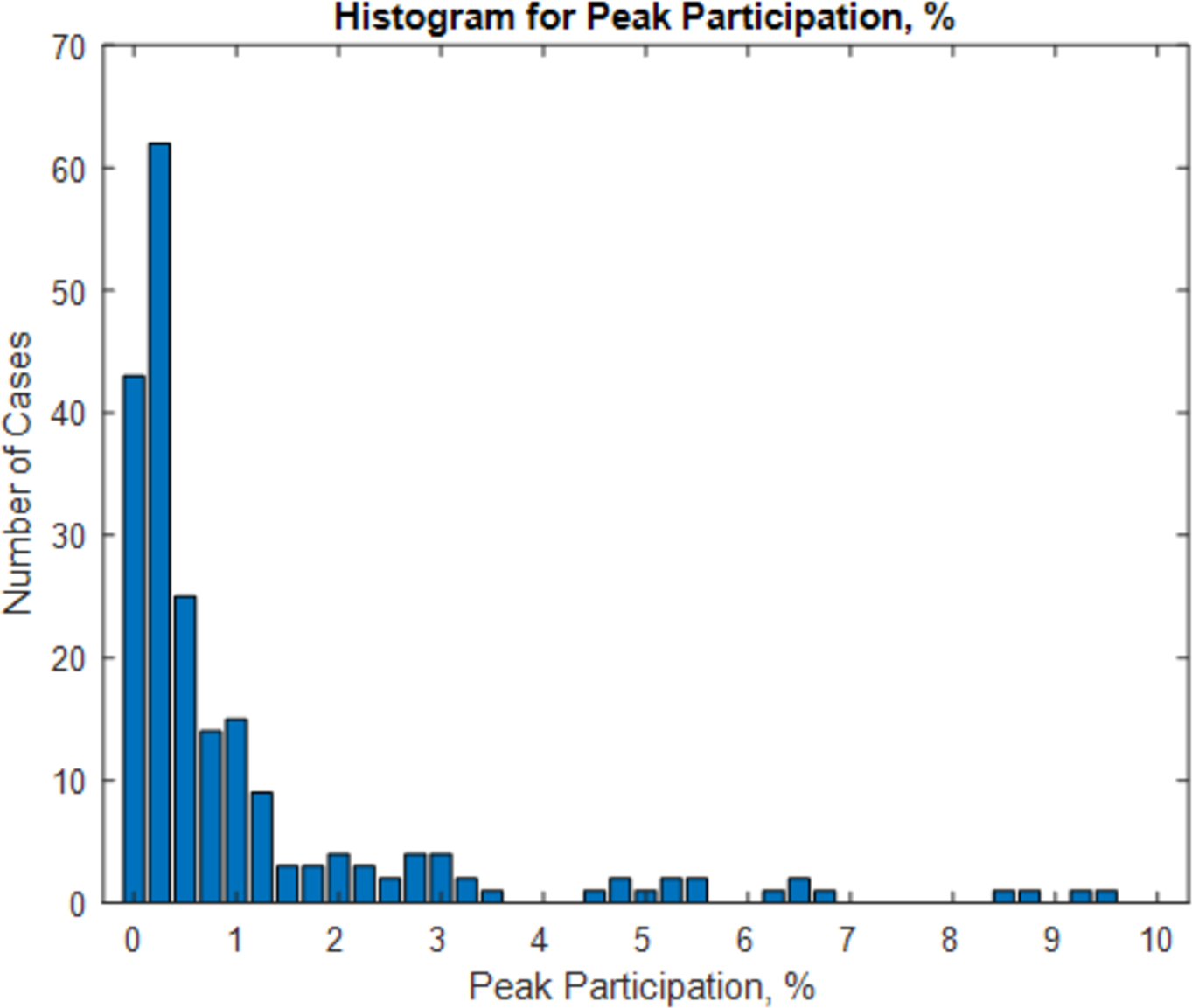

Supplement: S4 Fig — (TIF) [file pone.0269976.s005.tif]

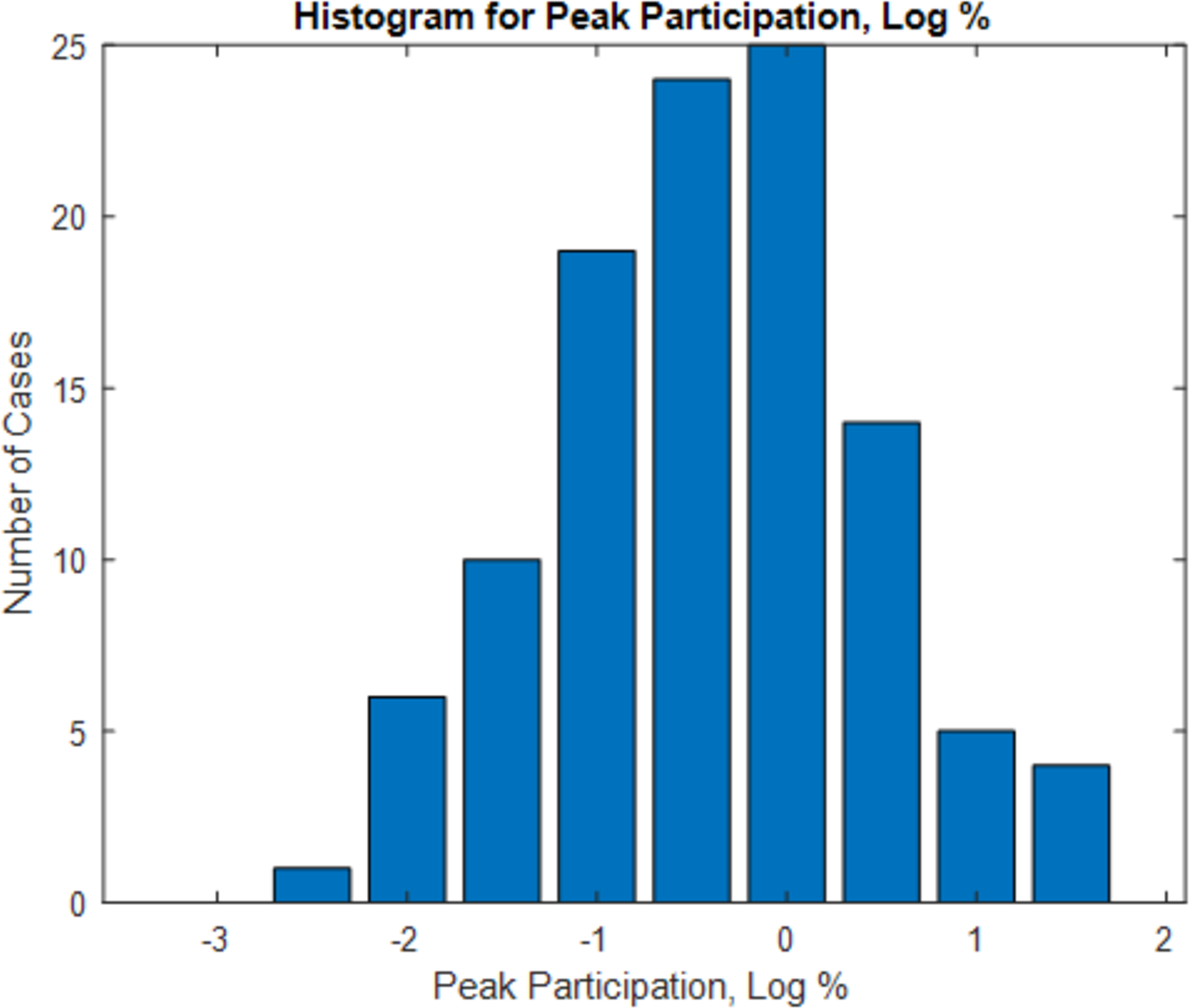

Supplement: S5 Fig — (TIF) [file pone.0269976.s006.tif]

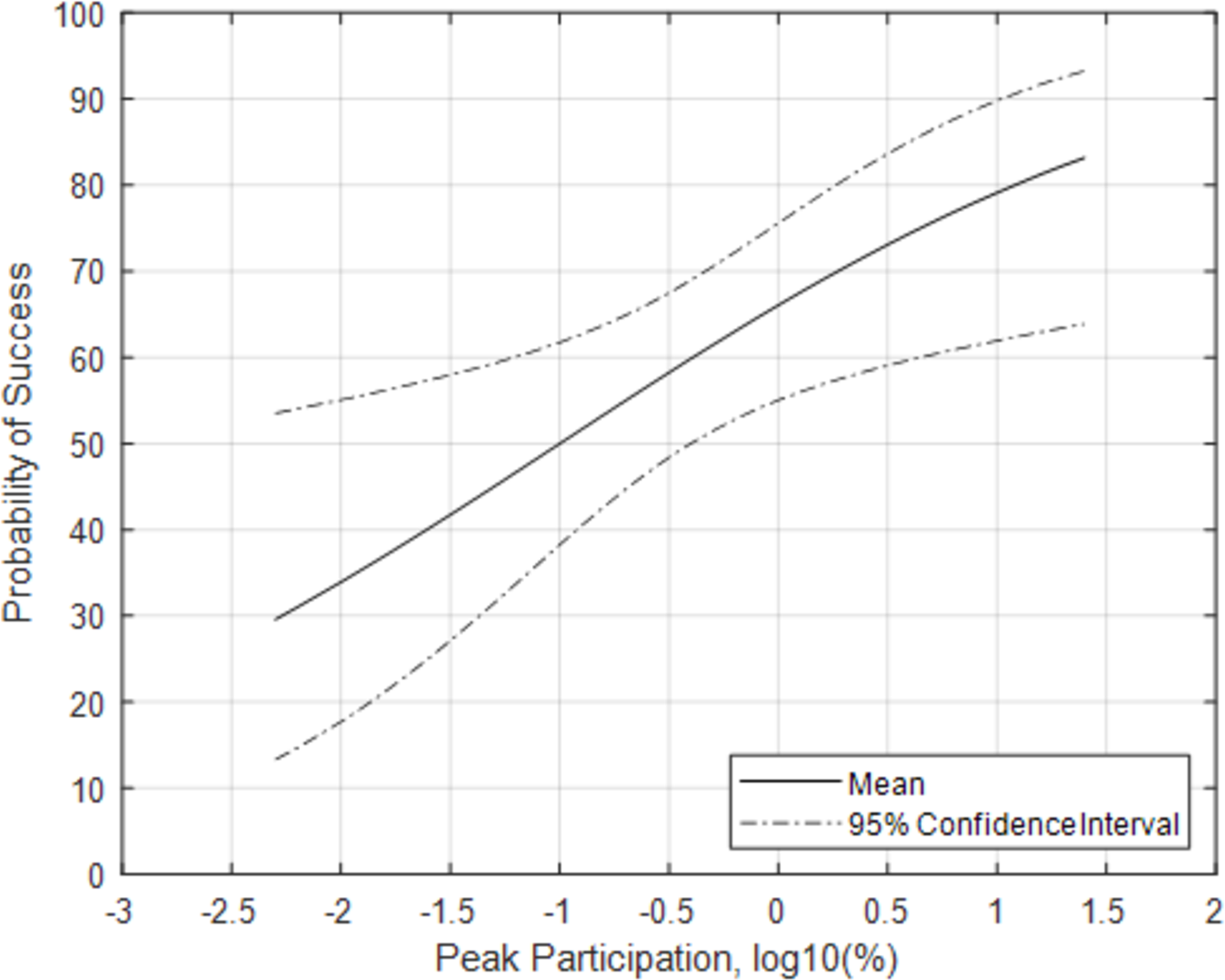

Supplement: S6 Fig — (TIF) [file pone.0269976.s007.tif]

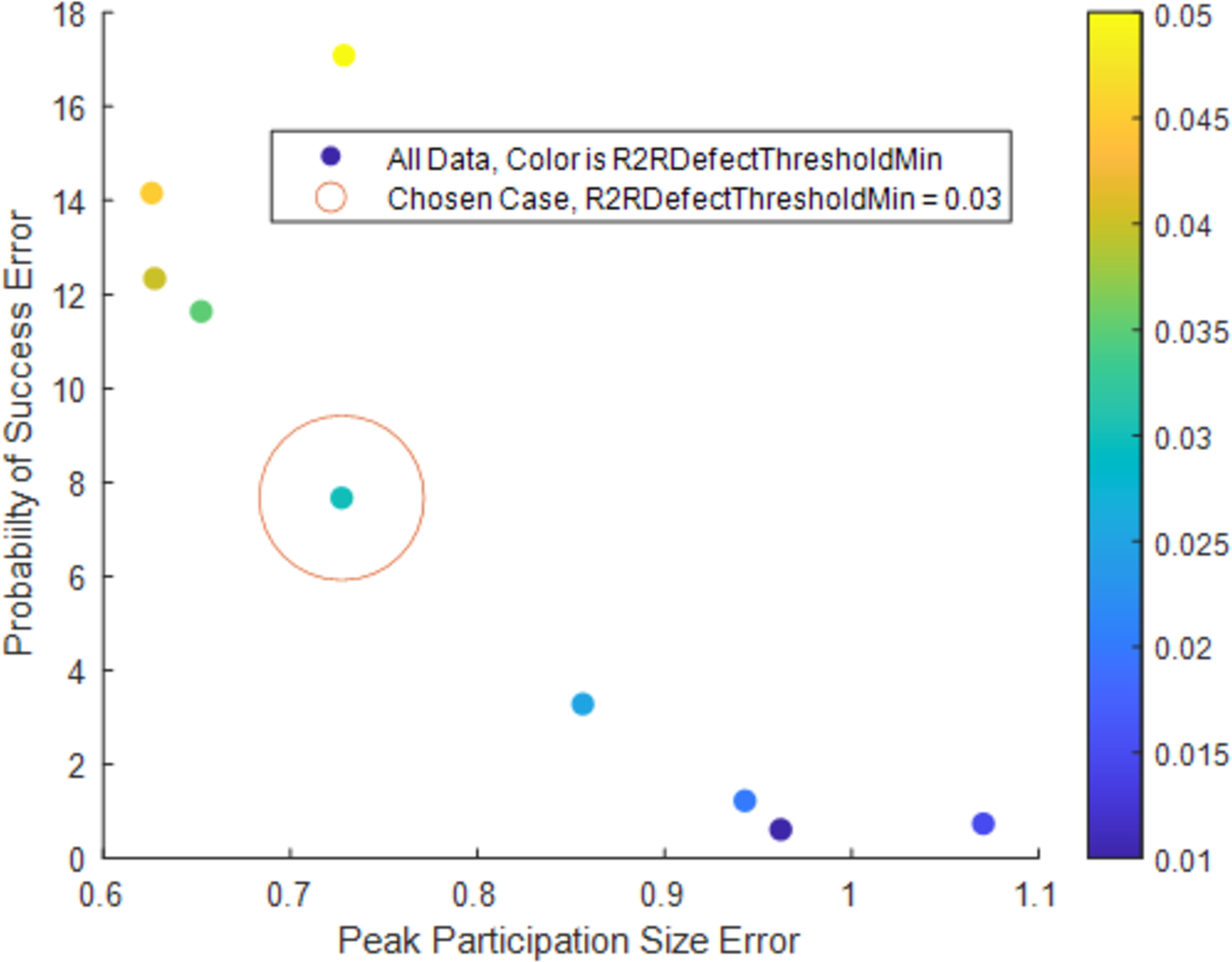

Supplement: S7 Fig — (TIF) [file pone.0269976.s008.tif]

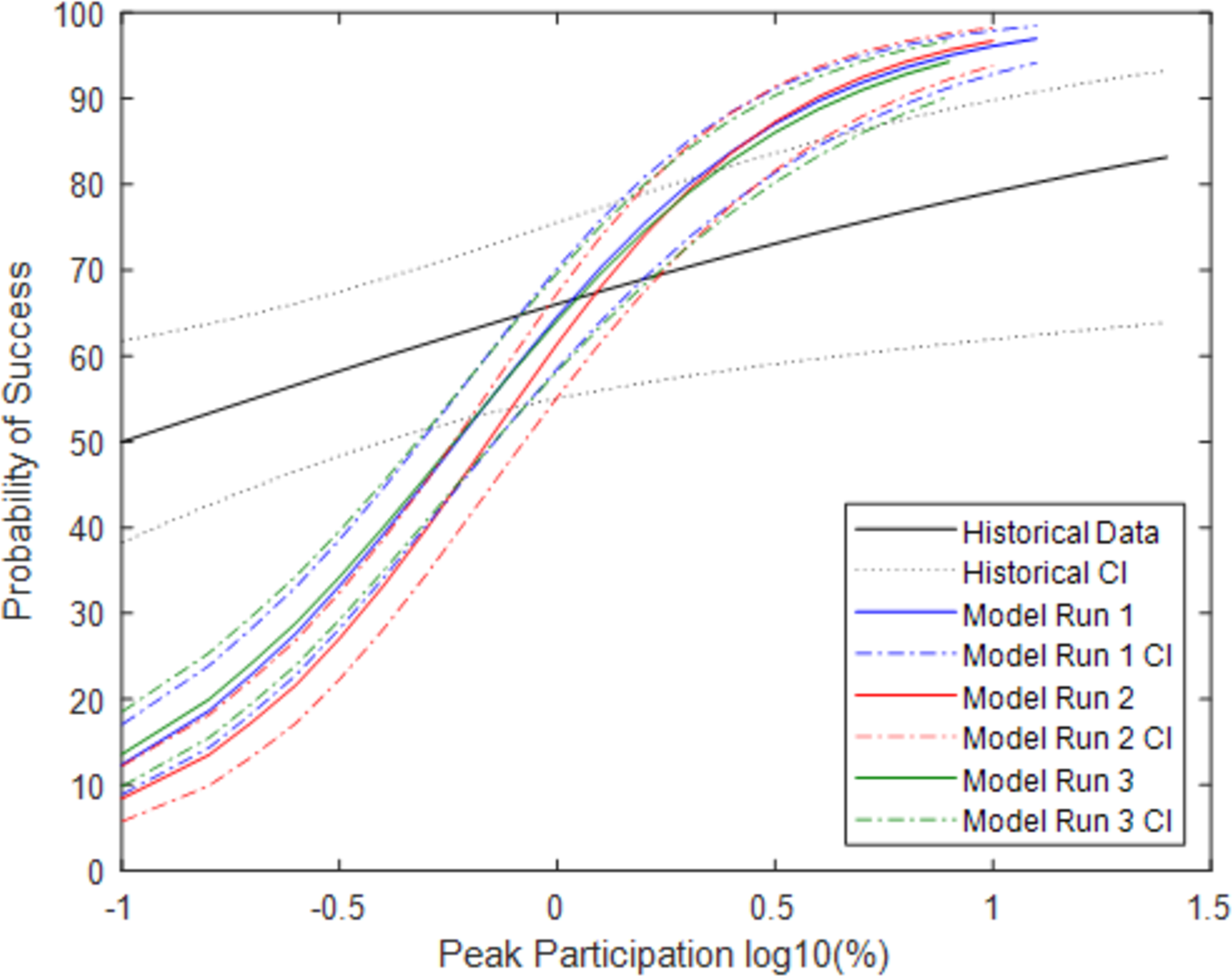

Supplement: S8 Fig — Error = 9.48, 13.0, and 8.7 respectively. (TIF) [file pone.0269976.s009.tif]

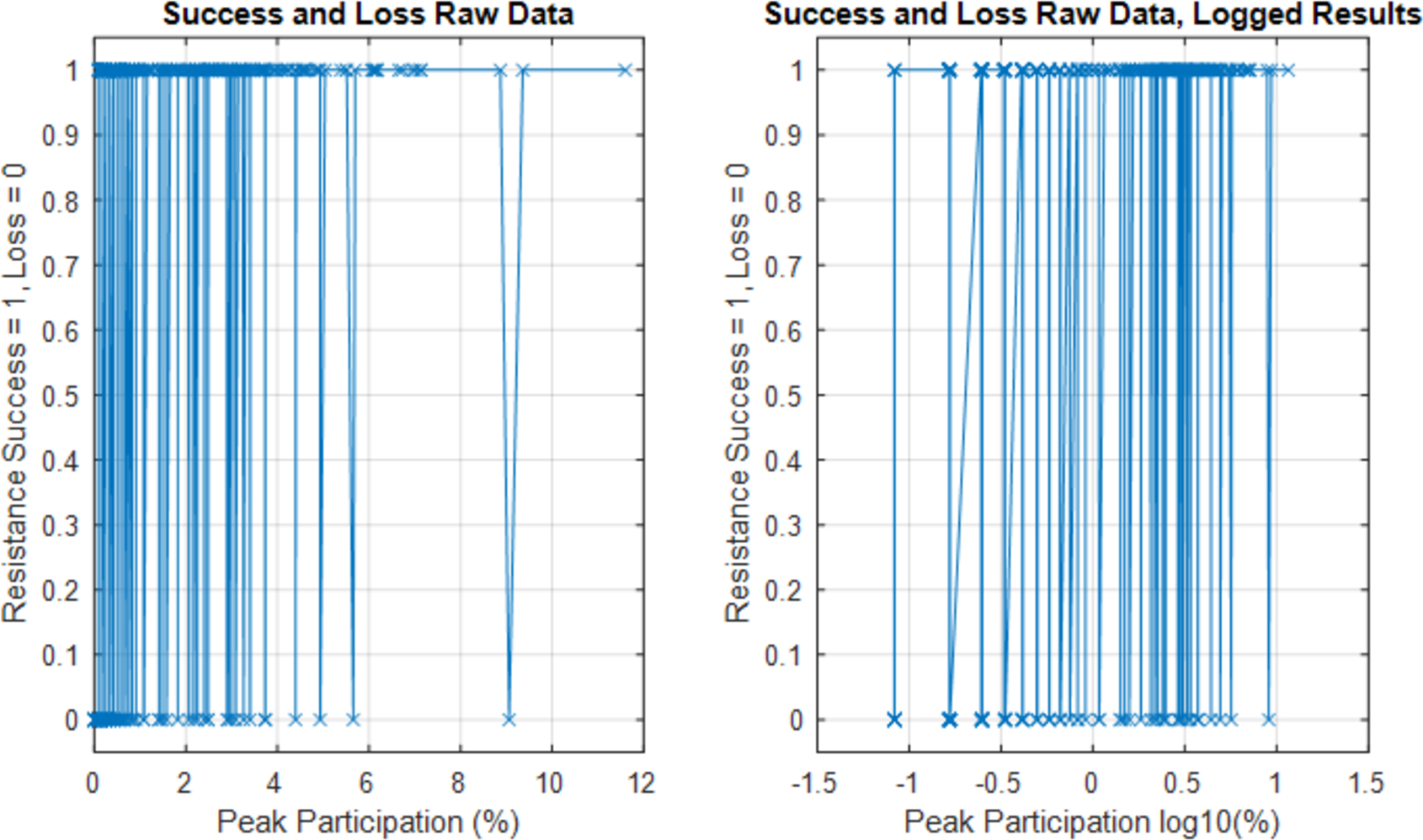

Supplement: S9 Fig — (TIF) [file pone.0269976.s010.tif]

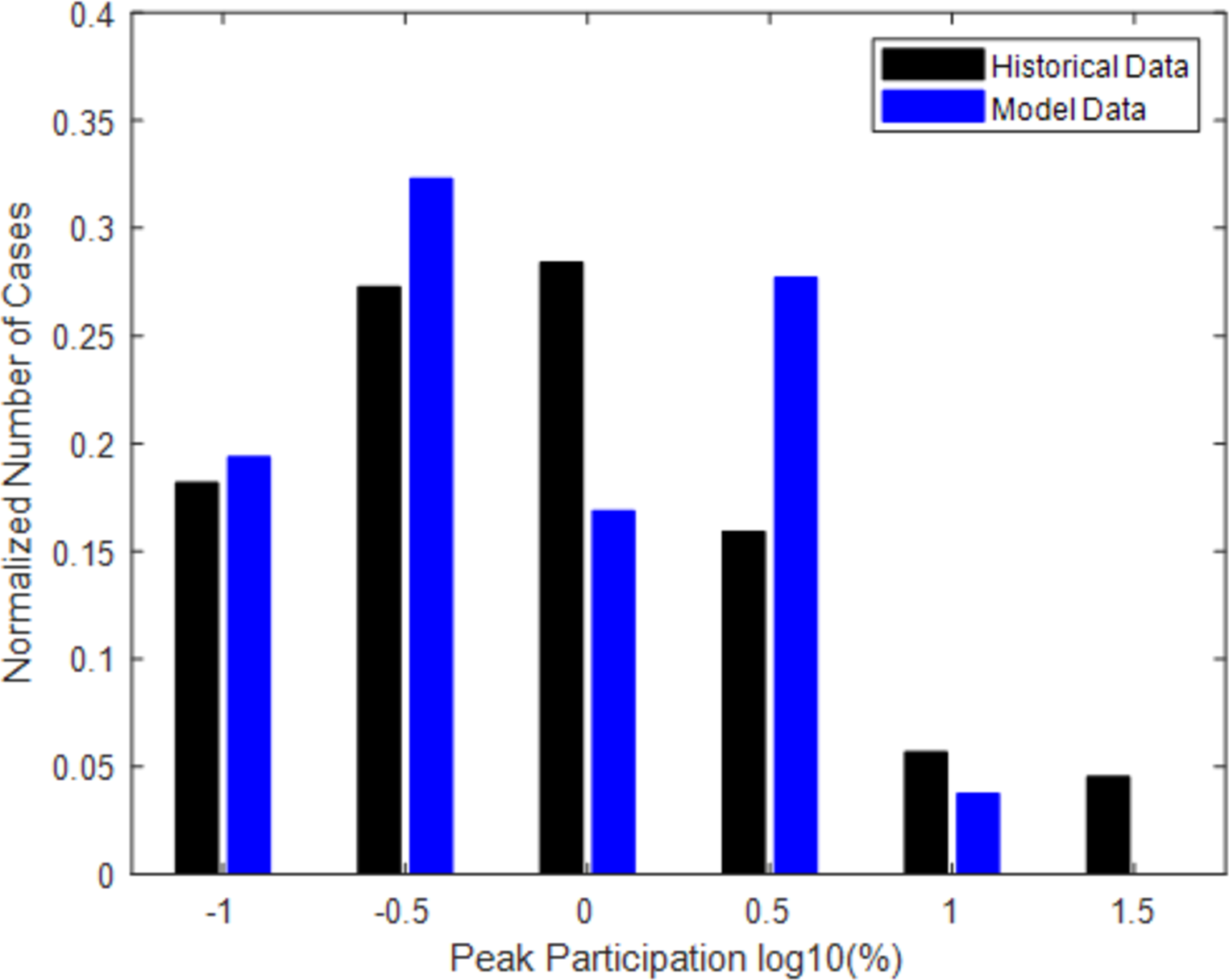

Supplement: S10 Fig — Error = 0.62. (TIF) [file pone.0269976.s011.tif]

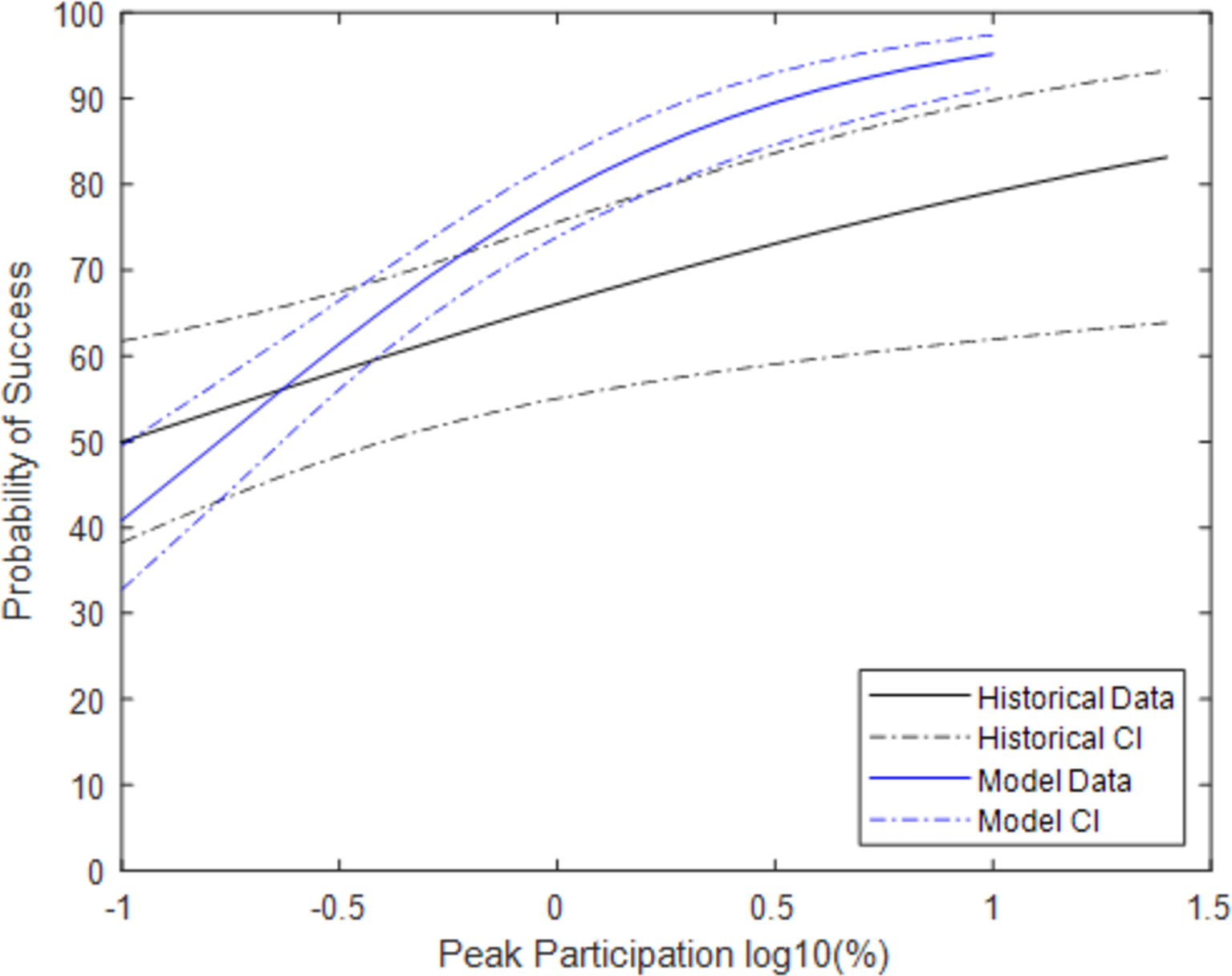

Supplement: S11 Fig — Error = 2.21. (TIF) [file pone.0269976.s012.tif]

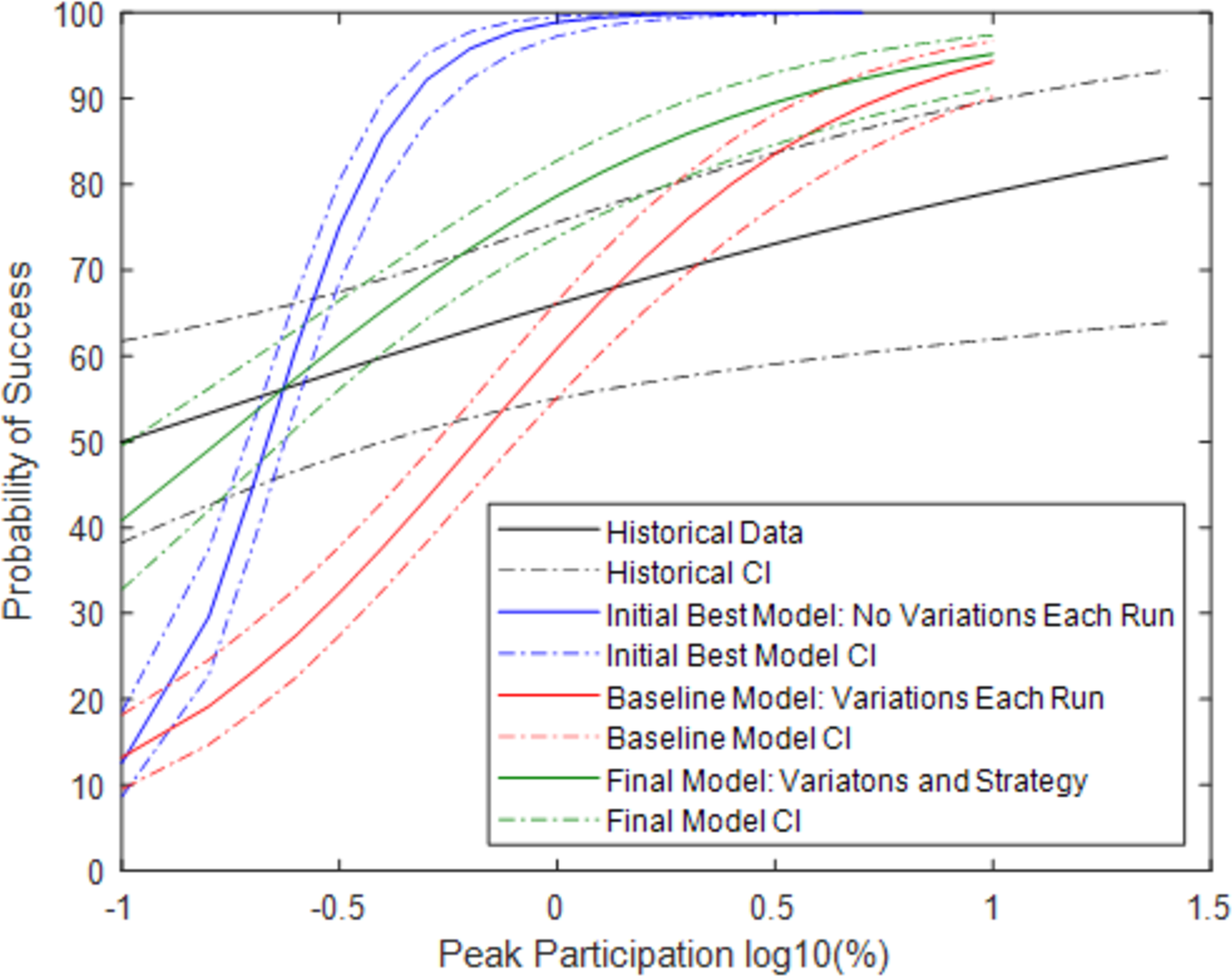

Supplement: S12 Fig — (TIF) [file pone.0269976.s013.tif]

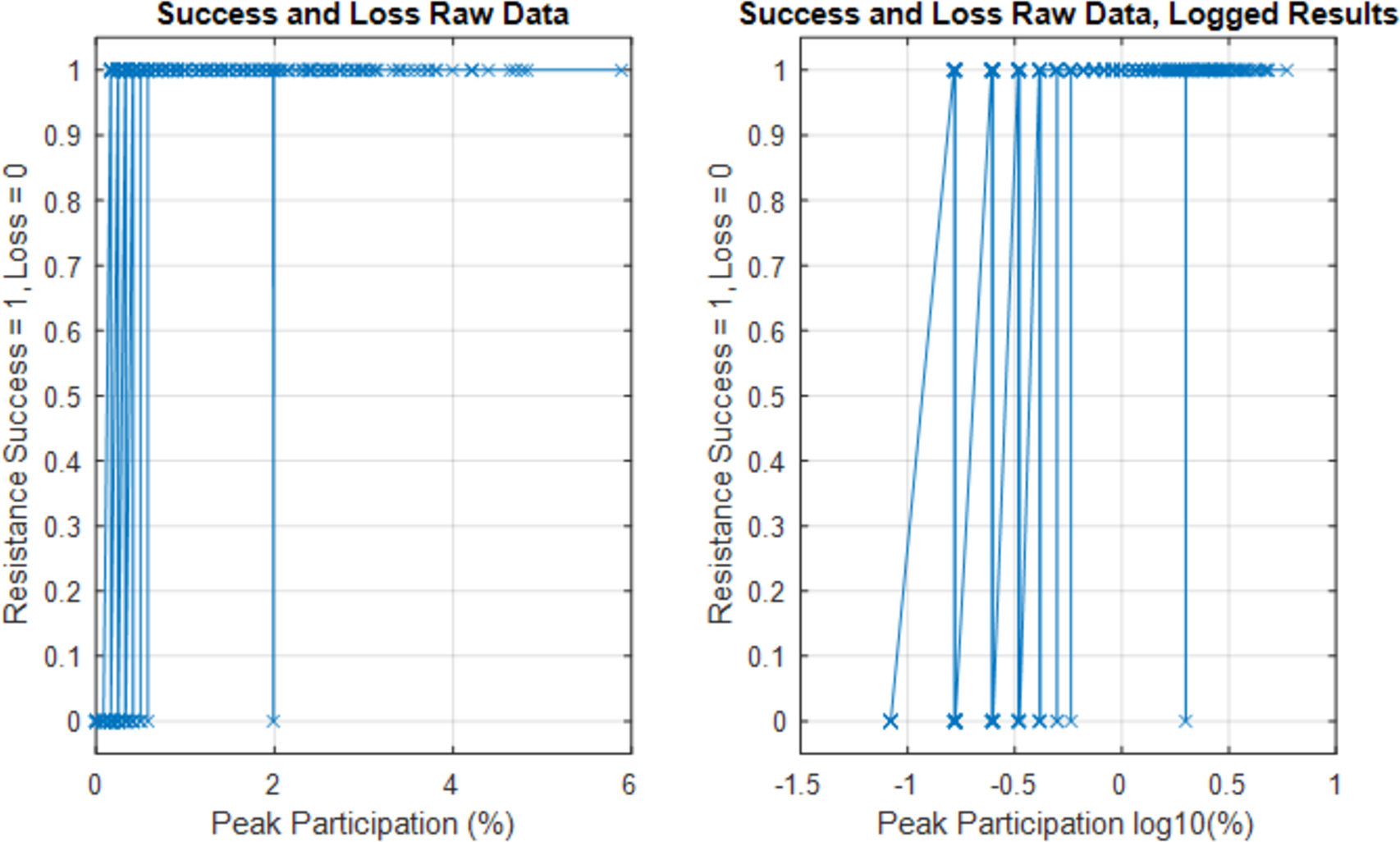

Supplement: S13 Fig — (TIF) [file pone.0269976.s014.tif]

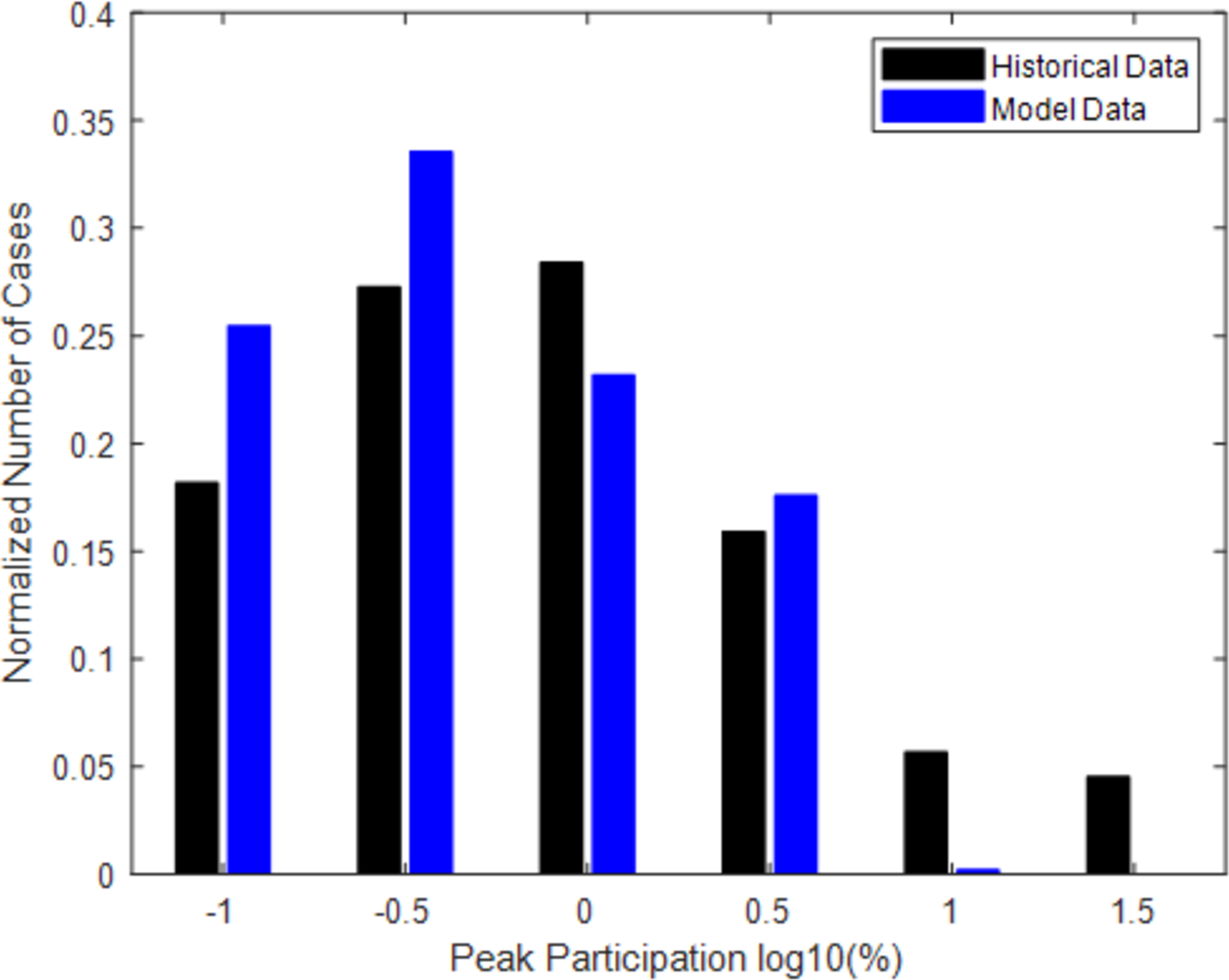

Supplement: S14 Fig — Error = 0.33. (TIF) [file pone.0269976.s015.tif]

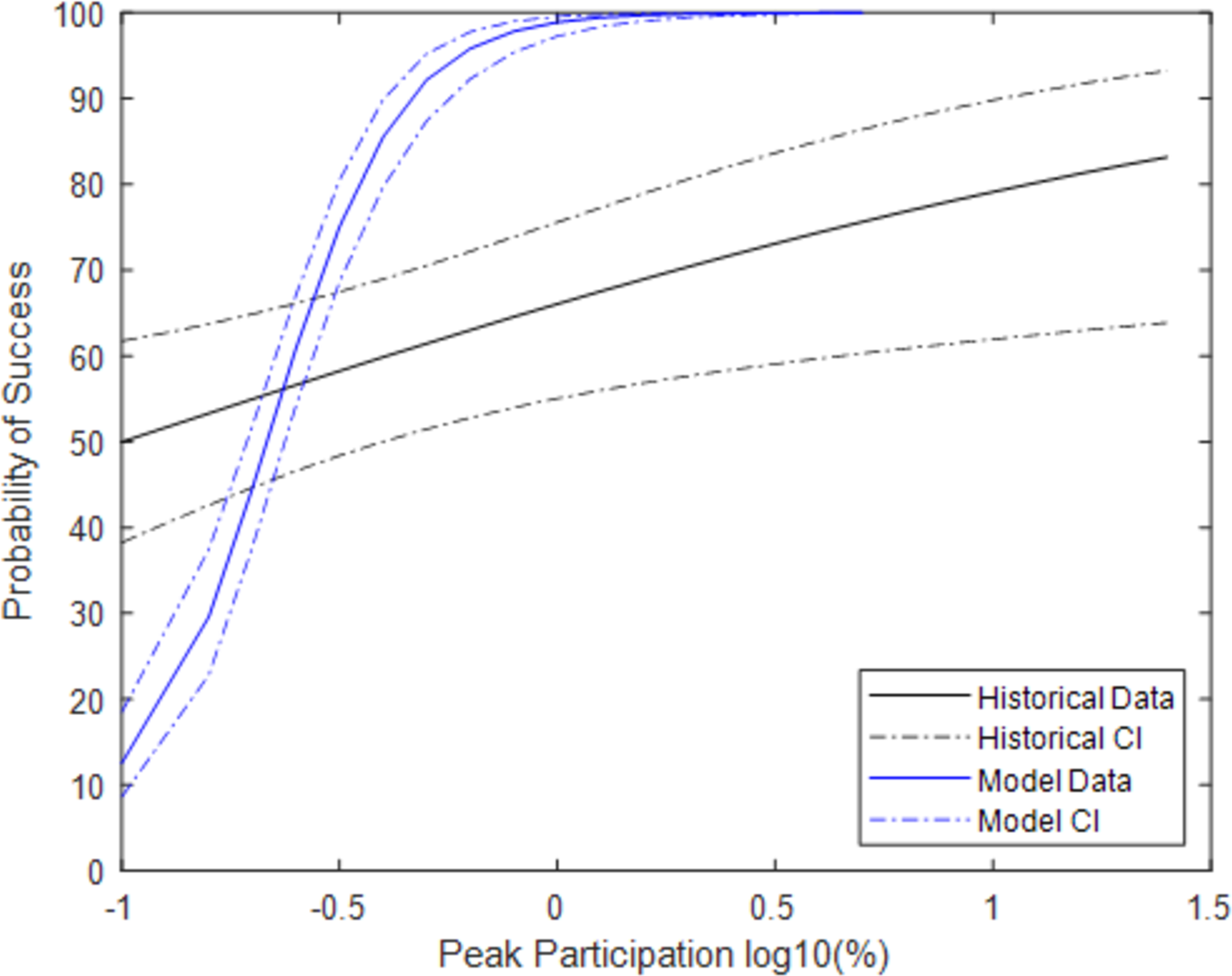

Supplement: S15 Fig — Error = 15.3. (TIF) [file pone.0269976.s016.tif]

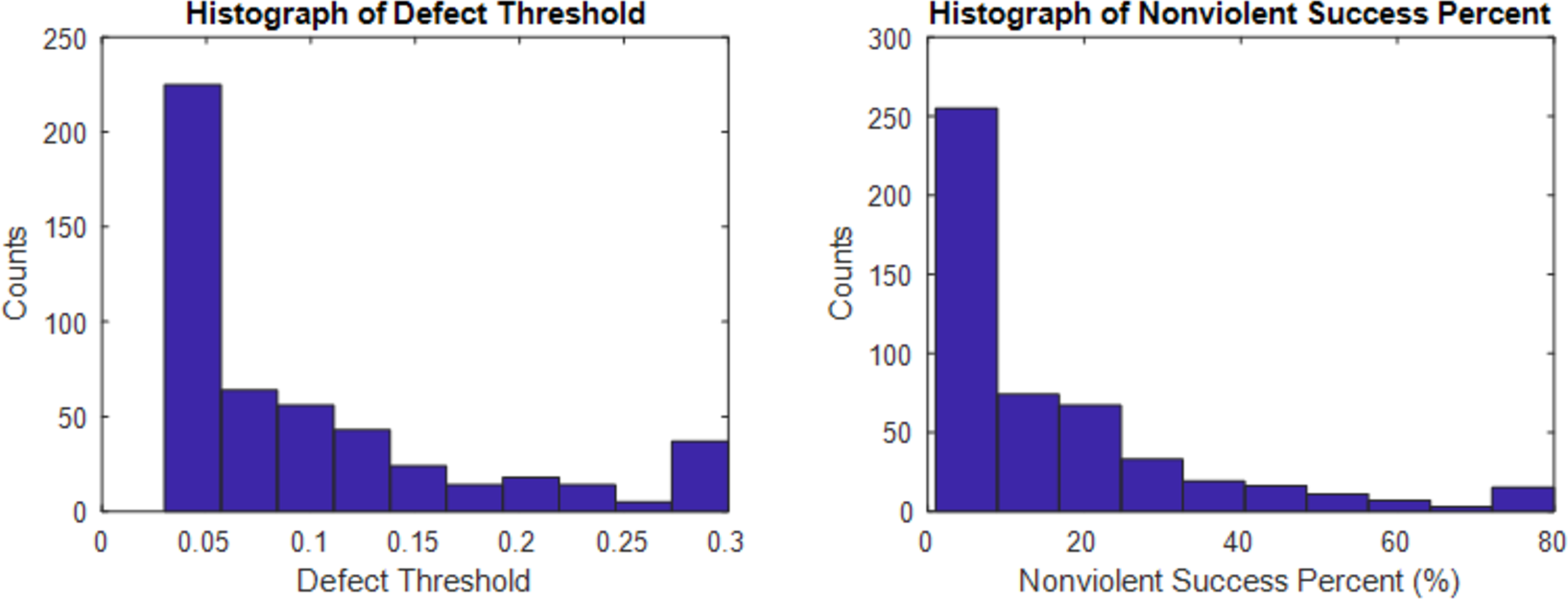

Supplement: S16 Fig — (TIF) [file pone.0269976.s017.tif]

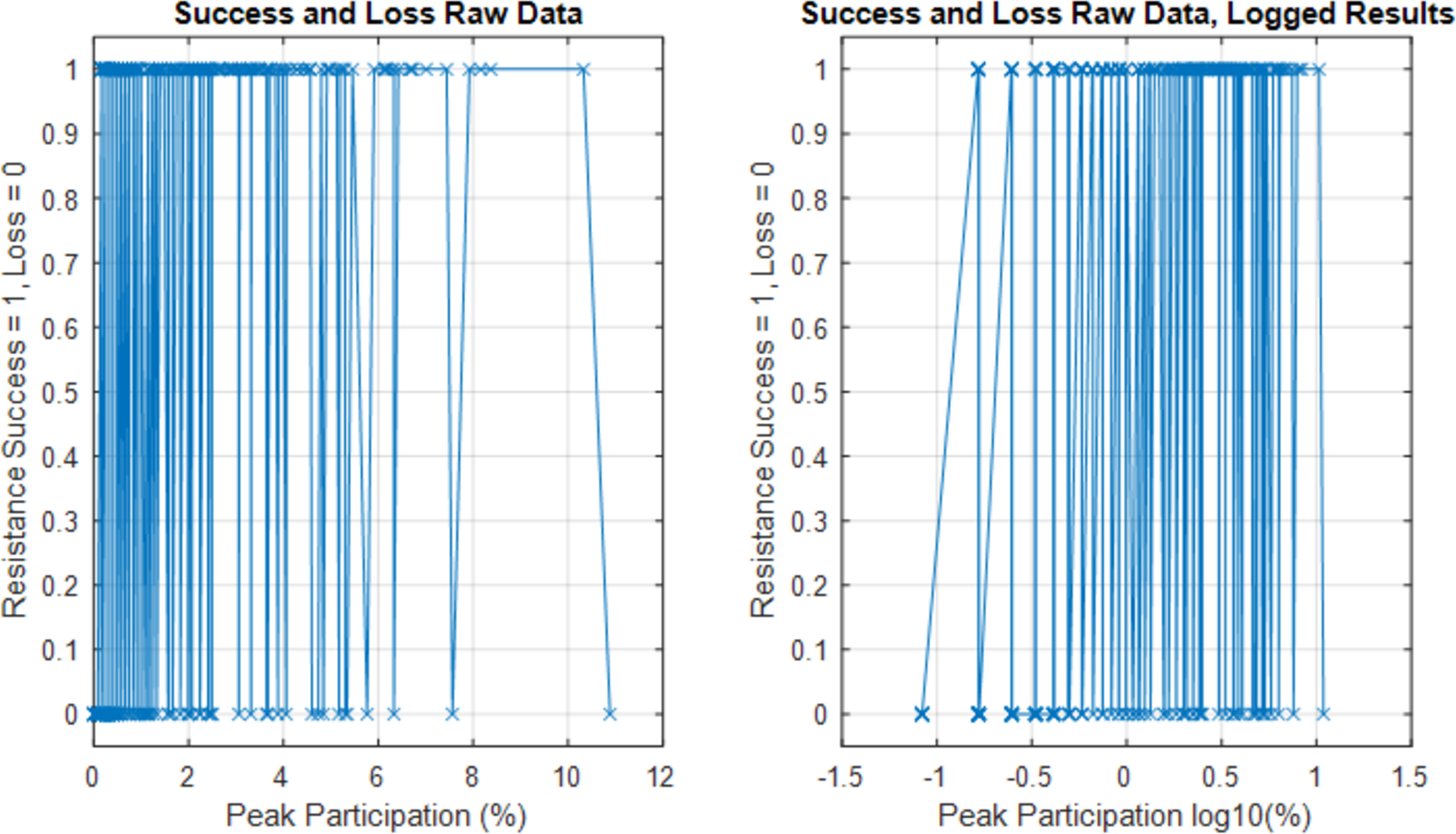

Supplement: S17 Fig — (TIF) [file pone.0269976.s018.tif]

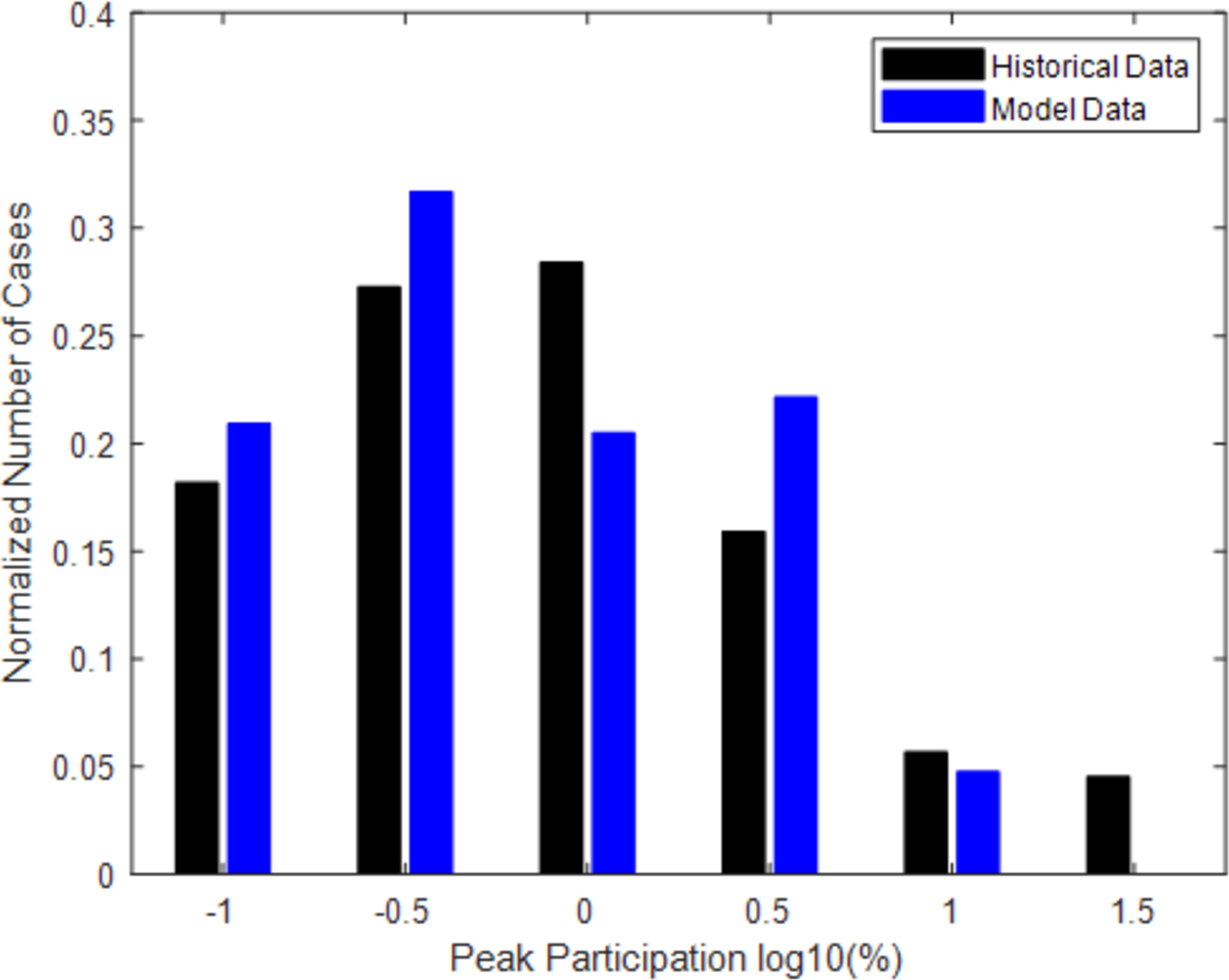

Supplement: S18 Fig — Error = 0.50. (TIF) [file pone.0269976.s019.tif]

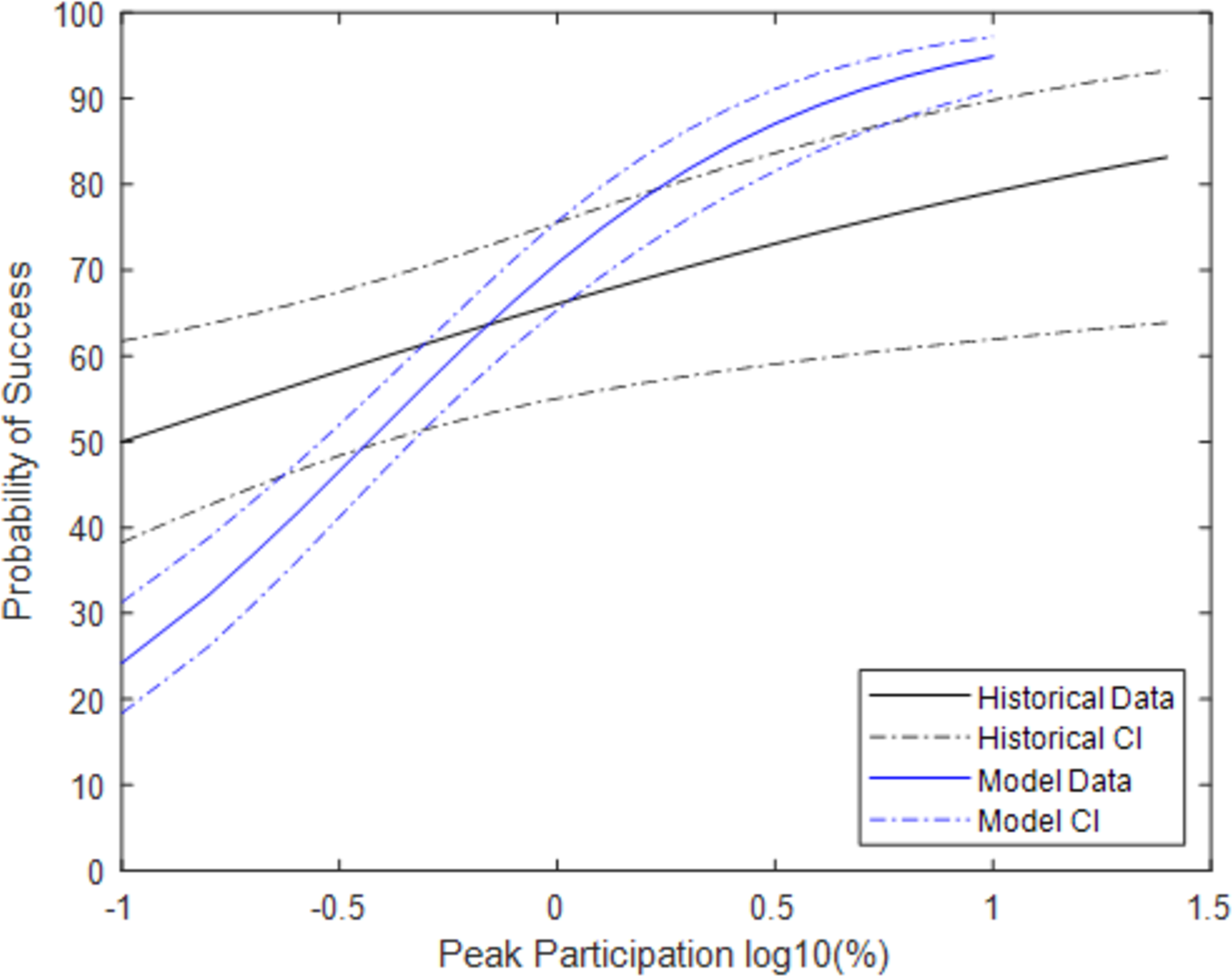

Supplement: S19 Fig — Error = 9.37. (TIF) [file pone.0269976.s020.tif]

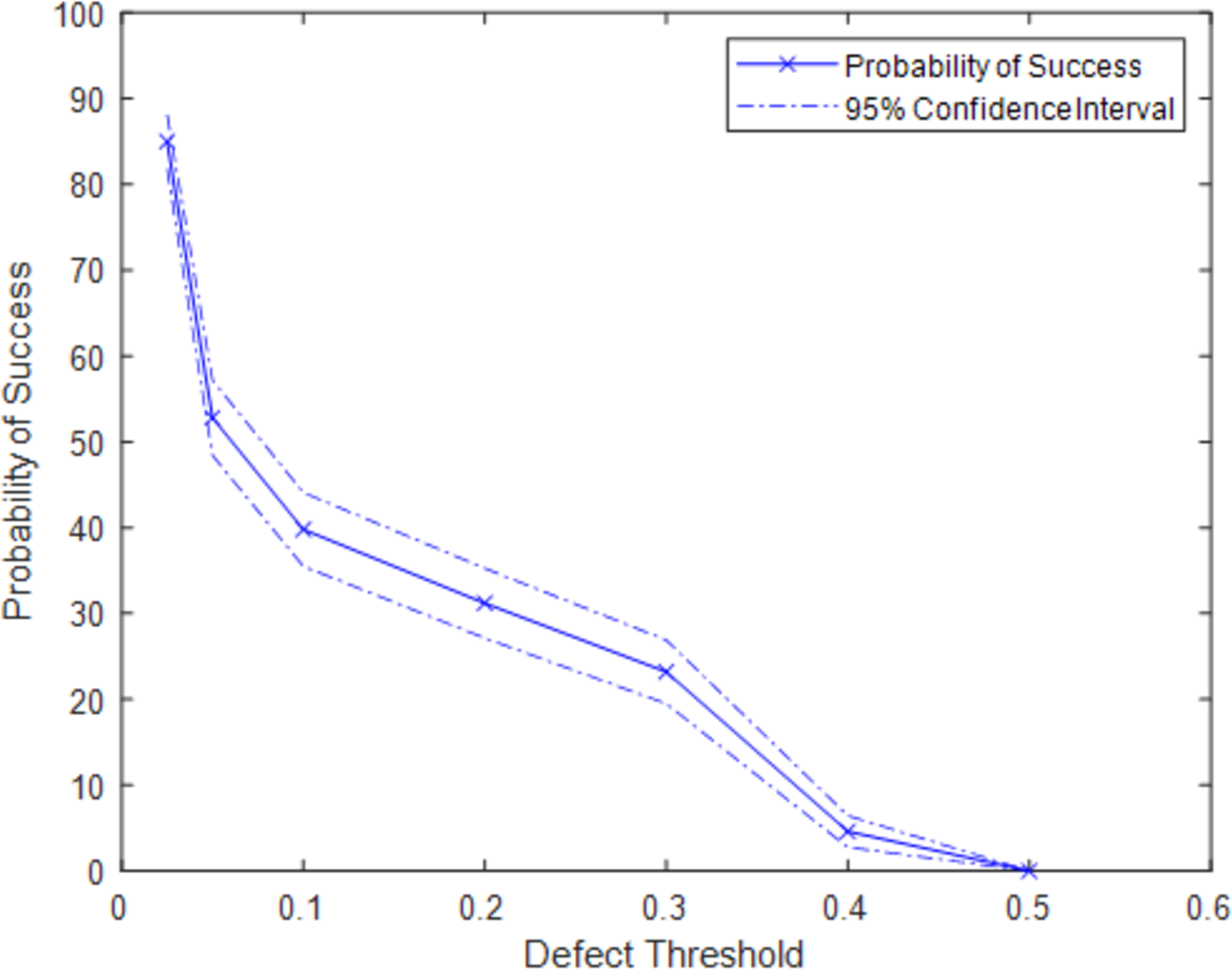

Supplement: S20 Fig — (TIF) [file pone.0269976.s021.tif]

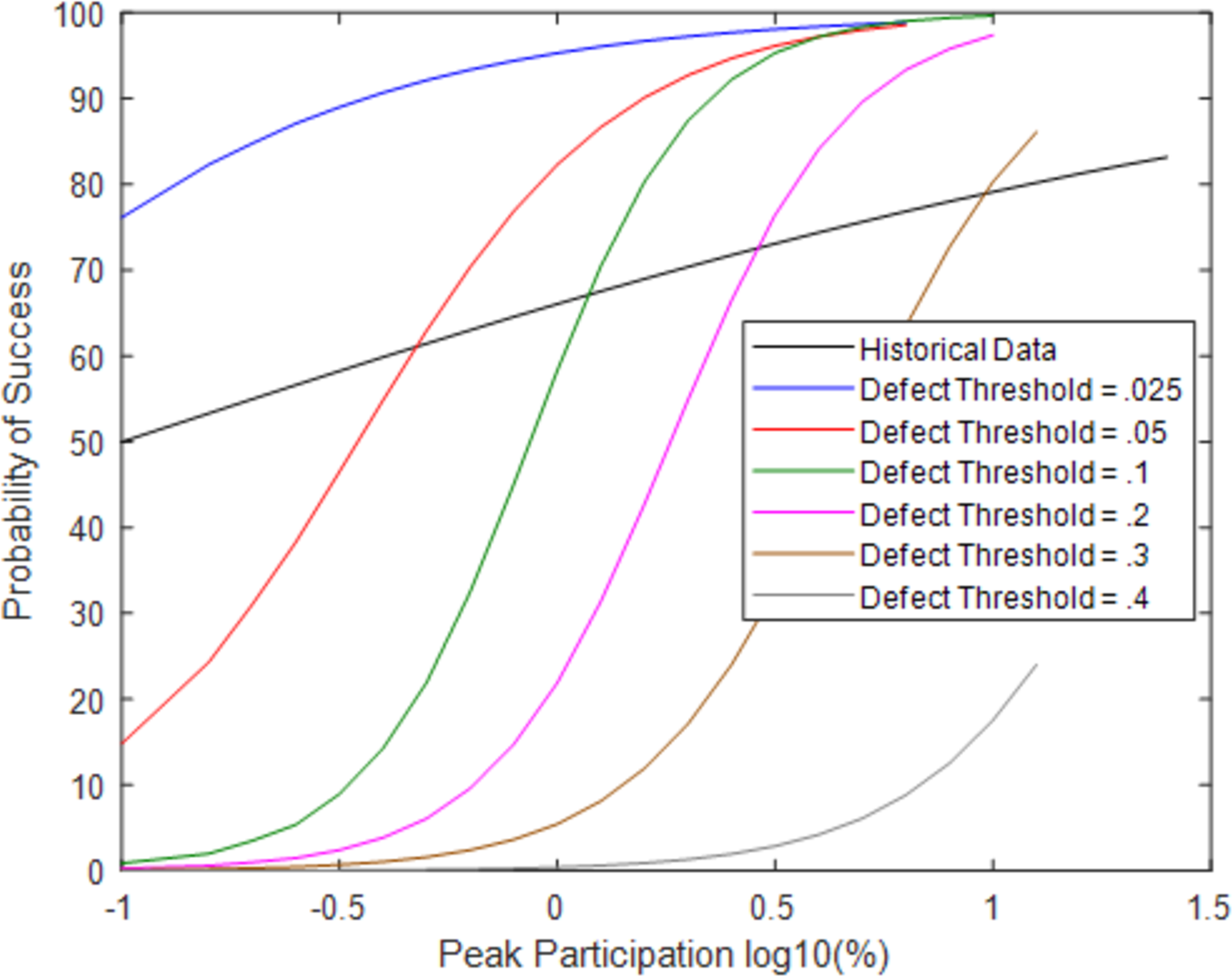

Supplement: S21 Fig — (TIF) [file pone.0269976.s022.tif]

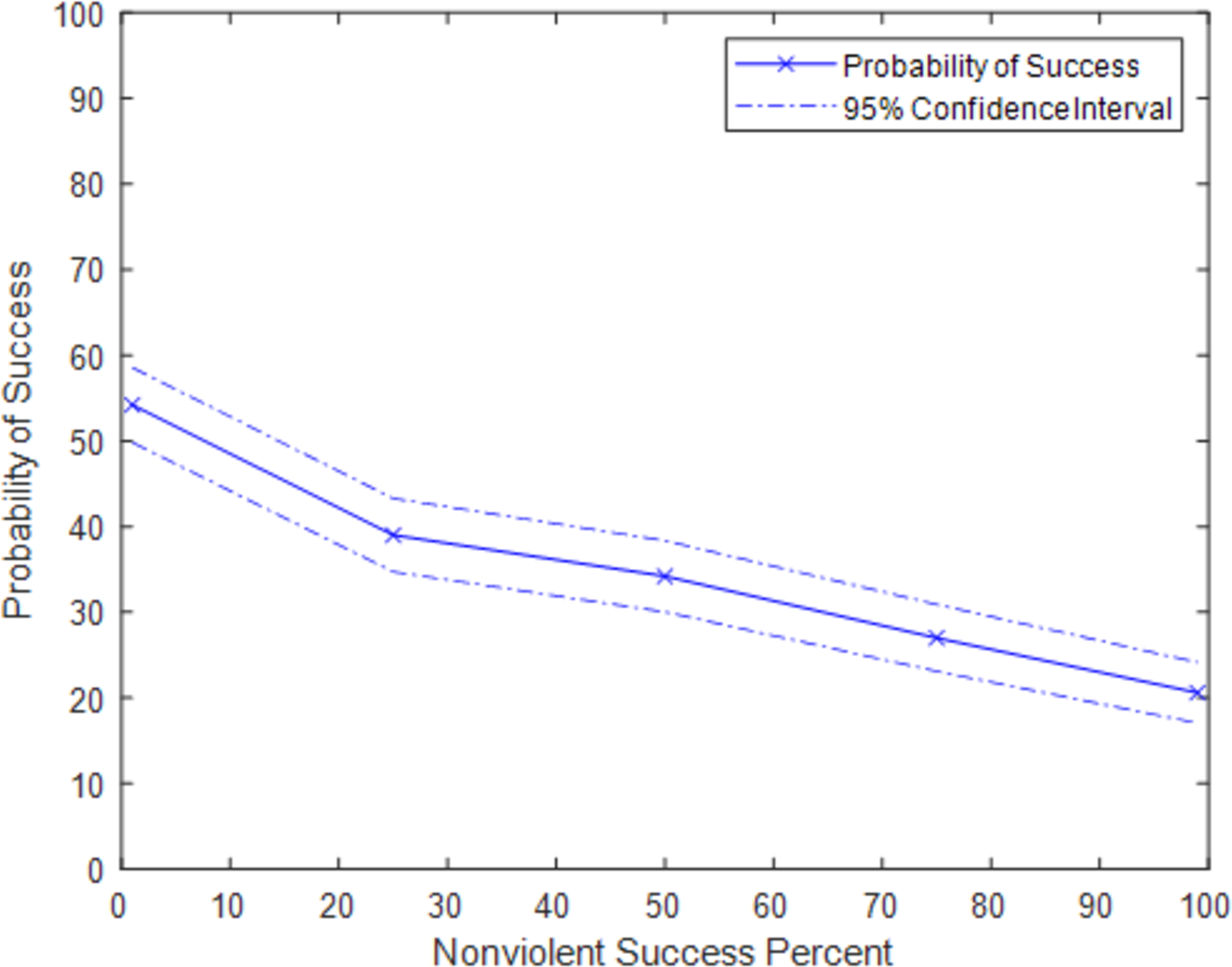

Supplement: S22 Fig — (TIF) [file pone.0269976.s023.tif]

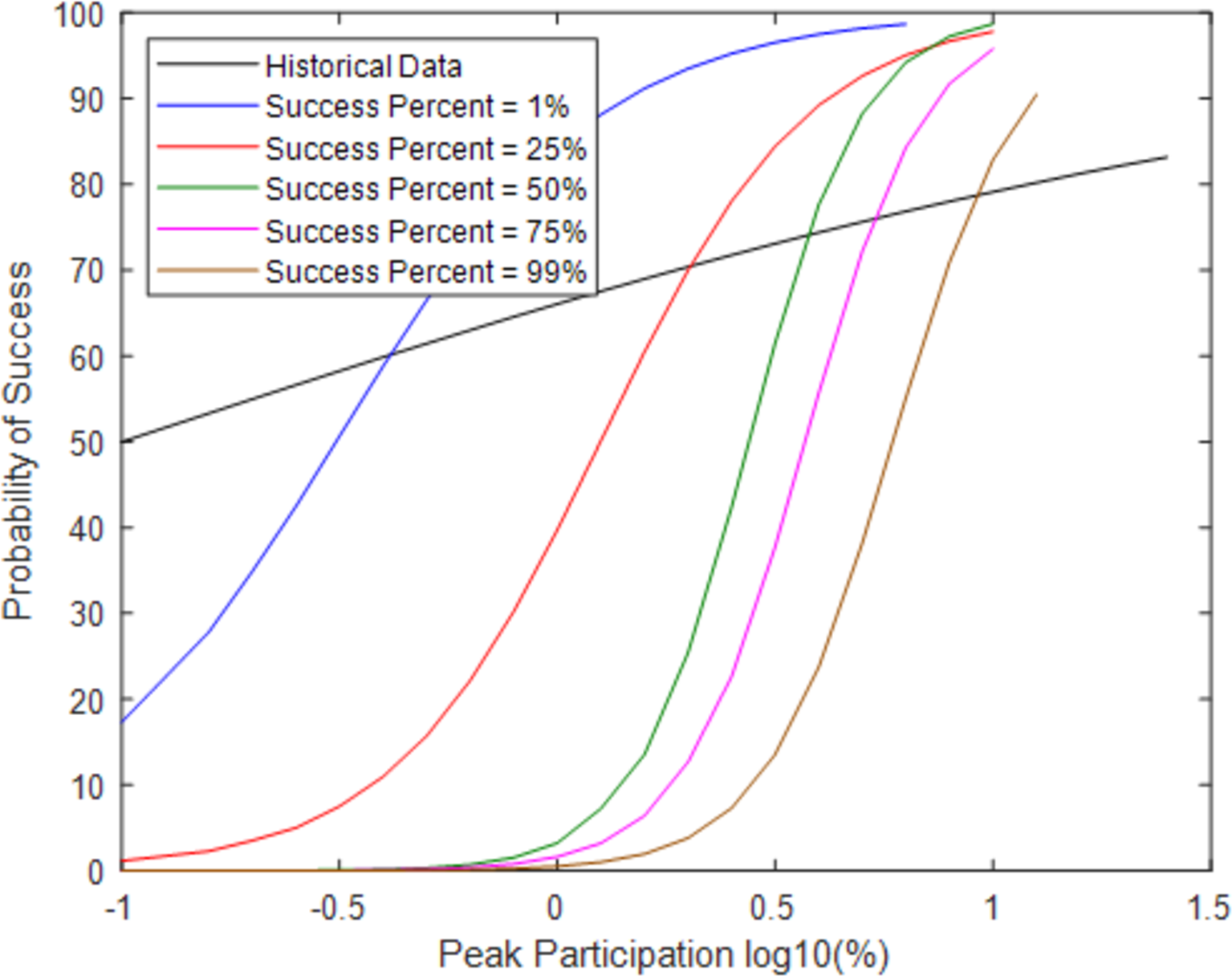

Supplement: S23 Fig — (TIF) [file pone.0269976.s024.tif]

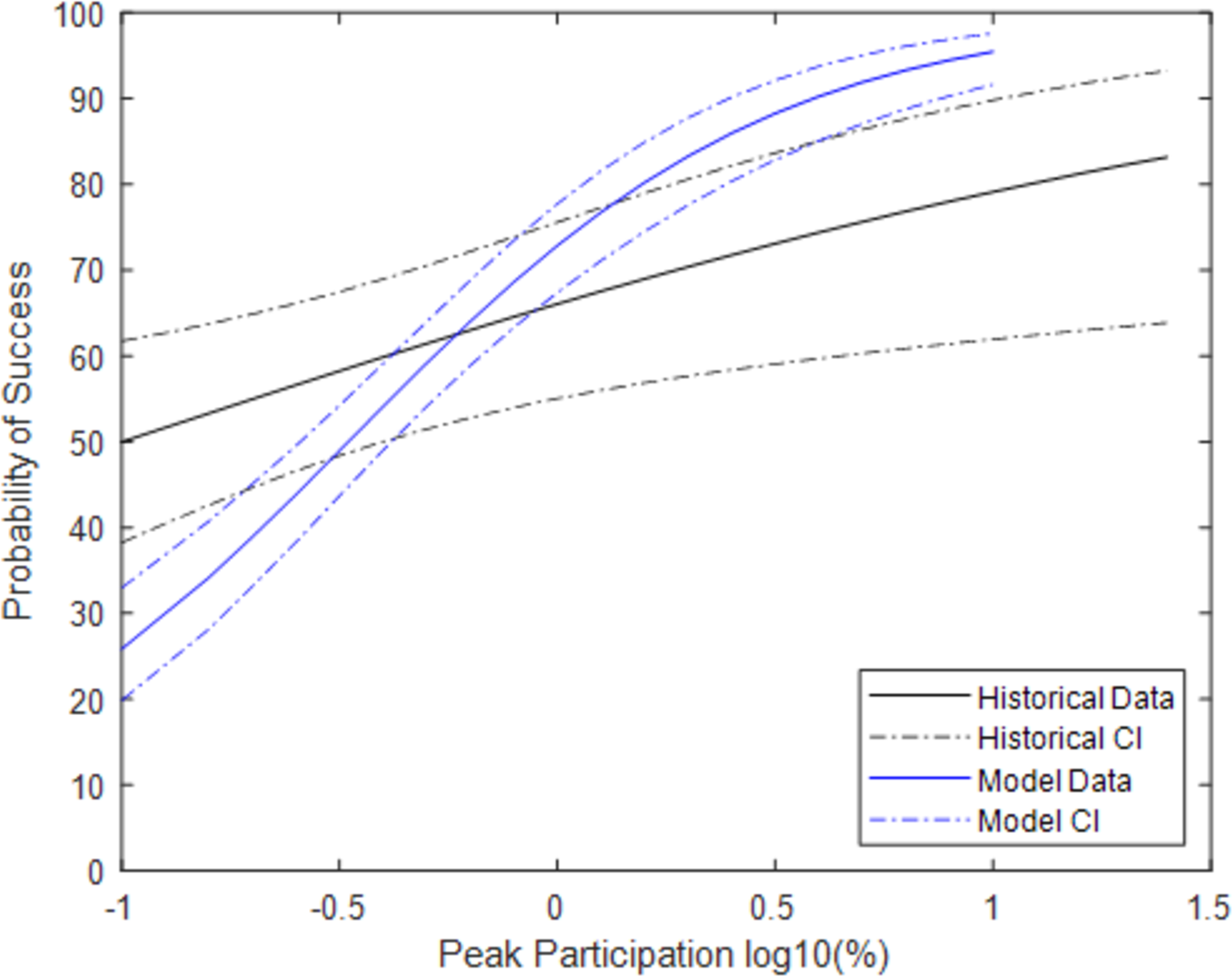

Supplement: S24 Fig — (TIF) [file pone.0269976.s025.tif]

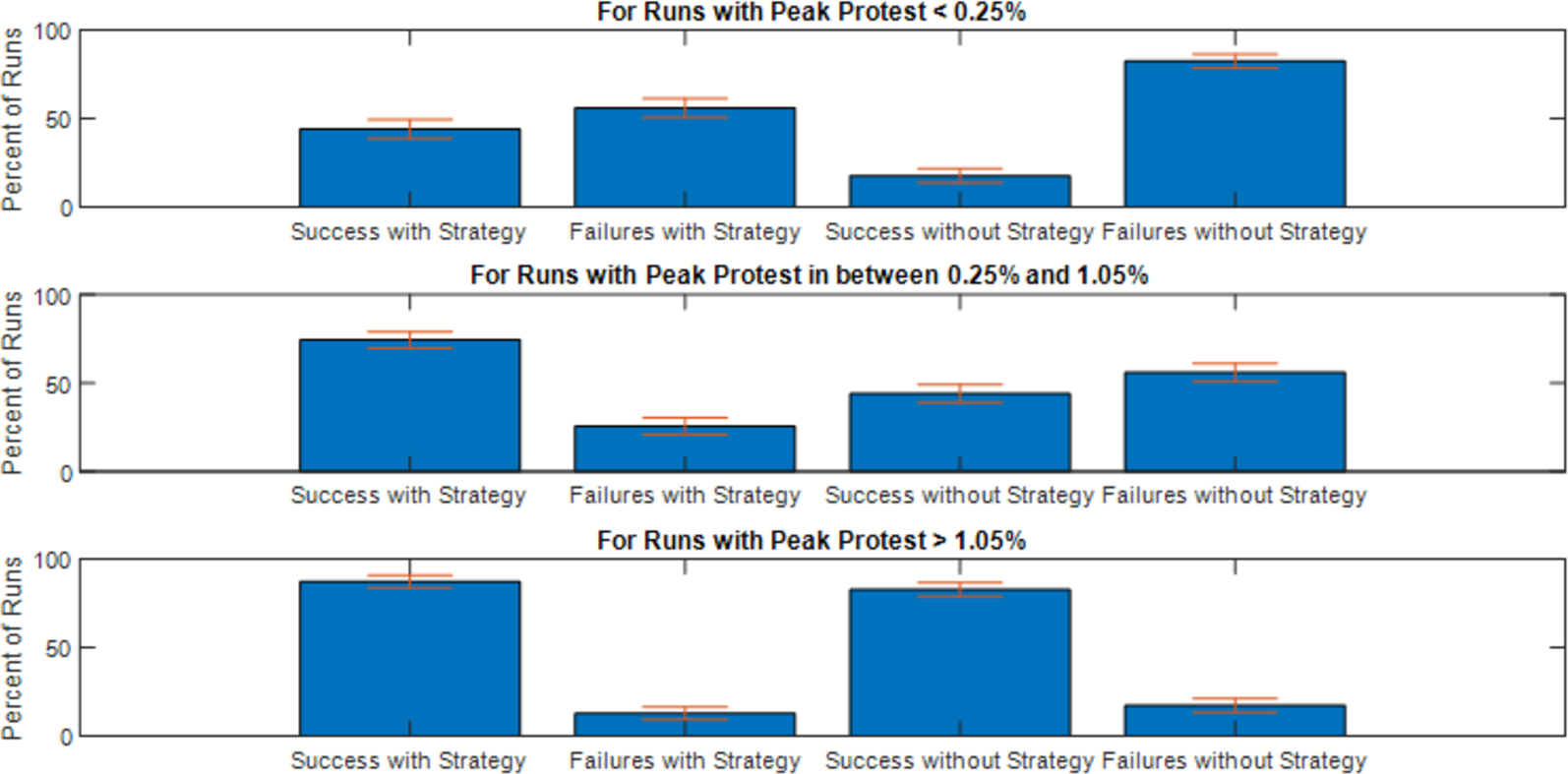

Supplement: S25 Fig — (TIF) [file pone.0269976.s026.tif]
