# Supplementary material for: A dynamic model of nonviolent resistance strategy
Source: PLoS One. 2022 Jul 27;17(7):e0269976. doi: 10.1371/journal.pone.0269976 (PMC9328538; doi:10.1371/journal.pone.0269976)
Supplement: S1 Table — *Note that density is not percentage of the populace, but percentage of allowable spaces in the torus lattice. **The MATLAB model uses a variable entitled Percent Fickle, whose value equals 100—Percent Committed. For the sake of clarity, this article uses the term Percent Committed. (DOCX) [file pone.0269976.s027.docx]

| **Variable** | **Typical Value/Range** | **Description** | **Rationale** |
| --- | --- | --- | --- |
| Max Steps | 200 time steps | Maximum number of time steps before simulation stops | Produces reasonable outputs |
| LatticeX | 40 spaces | Number of spaces in graph in X direction | From Moro model |
| LatticeY | 40 spaces | Number of spaces in graph in Y direction | From Moro model |
| Delay Start Max | 5 time steps | Maximum time steps before an agent can be active in the model | Reasonable starting point |
| Reorder Agent Param | 1 | Reorders agents, but keeps pillars at the end | Reasonable starting point |
| Percent Fill Civilians* | 70% | Percent Lattice is filled with agent type | From Moro model |
| Percent Fill Activists* | Varies Each Run | Percent Lattice is filled with agent type | Produces reasonable outputs |
| Percent Fill Police* | 4% | Percent Lattice is filled with agent type | Based on historical data of security forces by population size |
| Percent Fill Pillars* | 0.85% | Percent Lattice is filled with agent type | Produces reasonable outputs |
| vision | 4 spaces | Number of spaces an agent can see in each direction | Produces reasonable outputs |
| Starting Government Legitimacy | 0.56 | Starting value between 0 and 1 for government legitimacy | Produces reasonable outputs |
| Max Jail Term | 10 time steps | Max Jail Term | Based on the Moro model, but decreased for faster simulations. |
| f | .0706 | Threshold used for civilian rule | Produces reasonable outputs |
| Backfire Coefficient | 0.99 | Factor by which government legitimacy decreases | Produces reasonable outputs |
| Chance Find NV Resistor | 40% | Chance police can find a nonviolent agent who is not protesting | Reasonable starting point |
| Chance Target Nonviolent | 25% | Chance police will target a nonviolent agent | Reasonable starting point |
| Chance Kill Nonviolent | 10% | Chance police will kill a nonviolent agent | Reasonable starting point |
| Protest Cycle | 7 time steps | Frequency at which a nonviolent agent can protest | Reasonable starting point |
| Protest Duration | 1 time step | Number of time steps a nonviolent agent can protest continuously | Produces reasonable outputs |
| nNV | 1 | Number of nonviolent people needed to persuade agent to protest | Produces reasonable outputs |
| Peer Pressure Number | 3.3884 | Number of Protestors for Peer Pressure Term to equal .5 | Produces reasonable outputs |
| Percent Committed** | 0-100% | Percent of nonviolent civilians who remain in the resistance when they no longer see protestors | Produces reasonable outputs |
| Percent Immediate Protest | 0-100% | Percent of nonviolent civilians who protest immediately after they join resistance, i.e. same time step | Produces reasonable outputs |
| Defect Threshold | Randomized each run within chi2 distribution (from a min of 0.03 to a max of 0.3) | Mean defect threshold assigned to pillars and police | Produces reasonable outputs |
| Defect Threshold St Dev | 0 | Standard deviation defect threshold which allows different defect thresholds for individual pillars and police | Found to not help match data. Only needed when Pillar Prox Strategy = 2 |
| Nonviolent Success Percent | Randomized each run within chi2 distribution (from a min of 1 to a max of 80) | Percent of pillars required to defect for resistance success | Produces reasonable outputs |
| Pillar Prox Strategy | Varies Each Run (Set to 0, 1, or 2 for different activist strategies) | 0=agents randomly move like the other agents  1=agents move as close as possible to a pillar in their neighborhood  2=agents look for the pillar with the lowest individual defect threshold and move as close as possible to that pillar | Produces reasonable outputs |
| Activist Search Vision | 10 spaces | Number of spaces an activist can see in each direction when looking for pillars in Pillar Prox Strategy is 2 | Reasonable starting point |
