# Supplementary material for: A dynamic model of nonviolent resistance strategy
Source: PLoS One. 2022 Jul 27;17(7):e0269976. doi: 10.1371/journal.pone.0269976 (PMC9328538; doi:10.1371/journal.pone.0269976)
Supplement: S2 Table — (DOCX) [file pone.0269976.s028.docx]

|  | Designation | Experiment 1 | Experiment 2 | Experiment 3 | Experiment 4 | Experiment 5 | Experiment 6 |
| --- | --- | --- | --- | --- | --- | --- | --- |
|  | Description | Sweep Percent Immediate Protest | Sweep Percent Committed | Sweep Pillar Prox Strategy: 0,1, and 2 | Sweep Max Steps | Sweep R2R_DefectThresholdMin | Repeat Baseline Case |
|  | In Figures | 2, 3 | 4, 5 | 8 | S2 | S7 | S8 |
| Model Parameters | Max Steps | 200 | 200 | 200 | 150, 200, 250, 300 | 200 | 200 |
|  | LatticeX | 40 | 40 | 40 | 40 | 40 | 40 |
|  | LatticeY | 40 | 40 | 40 | 40 | 40 | 40 |
|  | torus | 1 | 1 | 1 | 1 | 1 | 1 |
|  | DelayStartMax | 5 | 5 | 5 | 5 | 5 | 5 |
|  | ReorderAgentsParam | 1 | 1 | 1 | 1 | 1 | 1 |
|  | PercentFillCivilians | 70 | 70 | 70 | 70 | 70 | 70 |
|  | PercentFillActivists | n/a | n/a | n/a | n/a | n/a | n/a |
|  | PercentFillPolice | 4 | 4 | 4 | 4 | 4 | 4 |
|  | PercentFillPillars | 0.85 | 0.85 | 0.85 | 0.85 | 0.85 | 0.85 |
|  | vision | 4 | 4 | 4 | 4 | 4 | 4 |
|  | MaxJailTerm | 10 | 10 | 10 | 10 | 10 | 10 |
|  | StartingGovernmentLegitimacy | 0.56 | 0.56 | 0.56 | 0.56 | 0.56 | 0.56 |
|  | ChanceFindNVResistor | 40 | 40 | 40 | 40 | 40 | 40 |
|  | ChanceTargetNonviolent | 25 | 25 | 25 | 25 | 25 | 25 |
|  | ChanceKillNonviolent | 10 | 10 | 10 | 10 | 10 | 10 |
|  | BackfireCoefficient | 0.99 | 0.99 | 0.99 | 0.99 | 0.99 | 0.99 |
|  | f | 0.0706 | 0.0706 | 0.0706 | 0.0706 | 0.0706 | 0.0706 |
|  | ProtestCycle | 7 | 7 | 7 | 7 | 7 | 7 |
|  | ProtestDuration | 1 | 1 | 1 | 1 | 1 | 1 |
|  | nNV | 1 | 1 | 1 | 1 | 1 | 1 |
|  | PeerPressureNumber | 3.3884 | 3.3884 | 3.3884 | 3.3884 | 3.3884 | 3.3884 |
|  | PercentCommitted | 0 | 0, 25, 50, 75, 100 | 0 | 0 | 0 | 0 |
|  | PercentImmediateProtest | 0, 25, 50, 75, 100 | 0 | 0 | 0 | 0 | 0 |
|  | DefectThreshold | n/a | n/a | n/a | n/a | n/a | n/a |
|  | DefectThresholdStDv | 0 | 0 | 0.01 | 0 | 0 | 0 |
|  | NVSuccessPercent | n/a | n/a | n/a | n/a | n/a | n/a |
|  | PillarProxStrategy | 0 | 0 | 0,1,2 | 0 | 0 | 0 |
|  | ActivistSearchVision | 10, unused | 10, unused | 10 | 10, unused | 10, unused | 10, unused |
| Run Parameters | R2R_PercentFillActivistMean | 0.8 | 0.8 | 0.8 | 0.8 | 0.8 | 0.8 |
|  | R2R_PercentFillActivistSTD | 0.3 | 0.3 | 0.3 | 0.3 | 0.3 | 0.3 |
|  | R2R_DefectThresholdMin | n/a | n/a | 0.03 | 0.03 | 0.01, 0.015, 0.045, 0.05 | 0.03 |
|  | R2R_DefectThresholdSTD | n/a | n/a | 0.07 | 0.07 | 0.07 | 0.07 |
|  | R2R_DefectThresholdMax | n/a | n/a | 0.3 | 0.3 | 0.3 | 0.3 |
|  | R2R_NVSuccessPercentMin | n/a | n/a | 1 | 1 | 1 | 1 |
|  | R2R_NVSuccessPercentSTD | n/a | n/a | 16 | 16 | 16 | 16 |
|  | R2R_NVSuccessPercentMax | n/a | n/a | 80 | 80 | 80 | 80 |
|  | R2R_PillarProxStrategyPercent | n/a | n/a | n/a | n/a | n/a | n/a |
| Results | Number of Runs | 500 | 500 | 500 | 500 | 500 | 500 |
|  | Participation Size Error | n/a | n/a | n/a | n/a | n/a | 0.70, 0.50 |
|  | Probability of Success Error | n/a | n/a | n/a | n/a | n/a | 9.48, 13.0 |
|  | Success Percentage | 50%, 60%, 70%, 76%, 80%, | 49%, 55%, 54%, 59%, 62% | 48%, 68%, 88% | n/a | n/a | 47%, 44%, 48% |
|  | Case with Most Success | Immediate Protest  = 100% | Committed = 100% | Pillar Strategy  = 2 | n/a | n/a | n/a |
