# Supplementary material for: A dynamic model of nonviolent resistance strategy
Source: PLoS One. 2022 Jul 27;17(7):e0269976. doi: 10.1371/journal.pone.0269976 (PMC9328538; doi:10.1371/journal.pone.0269976)
Supplement: S3 Table — (DOCX) [file pone.0269976.s029.docx]

|  | Designation | Model 4 | Model 1 | Model 2 | Experiment 7 | Experiment 8 | Model 3 |
| --- | --- | --- | --- | --- | --- | --- | --- |
|  | Description | Best Fit | Initial Optimization | Baseline | Sweep Defect Threshold | Sweep Nonviolent Success Percent | Pillar Prox Strategy 50% |
|  | In Figures | S9, S10, S11, S12 | S12, S13, S14, S15 | S12, S17, S18, S19 | S20, S21 | S22, S23 | S24, S25 |
| Model Parameters | Max Steps | 200 | 200 | 200 | 200 | 200 | 200 |
|  | LatticeX | 40 | 40 | 40 | 40 | 40 | 40 |
|  | LatticeY | 40 | 40 | 40 | 40 | 40 | 40 |
|  | torus | 1 | 1 | 1 | 1 | 1 | 1 |
|  | DelayStartMax | 5 | 5 | 5 | 5 | 5 | 5 |
|  | ReorderAgentsParam | 1 | 1 | 1 | 1 | 1 | 1 |
|  | PercentFillCivilians | 70 | 70 | 70 | 70 | 70 | 70 |
|  | PercentFillActivists | n/a | n/a | n/a | n/a | n/a | n/a |
|  | PercentFillPolice | 4 | 4 | 4 | 4 | 4 | 4 |
|  | PercentFillPillars | 0.85 | 0.85 | 0.85 | 0.85 | 0.85 | 0.85 |
|  | vision | 4 | 4 | 4 | 4 | 4 | 4 |
|  | MaxJailTerm | 10 | 10 | 10 | 10 | 10 | 10 |
|  | StartingGovernmentLegitimacy | 0.56 | 0.56 | 0.56 | 0.56 | 0.56 | 0.56 |
|  | ChanceFindNVResistor | 40 | 40 | 40 | 40 | 40 | 40 |
|  | ChanceTargetNonviolent | 25 | 25 | 25 | 25 | 25 | 25 |
|  | ChanceKillNonviolent | 10 | 10 | 10 | 10 | 10 | 10 |
|  | BackfireCoefficient | 0.99 | 0.99 | 0.99 | 0.99 | 0.99 | 0.99 |
|  | f | 0.0706 | 0.0706 | 0.0706 | 0.0706 | 0.0706 | 0.0706 |
|  | ProtestCycle | 7 | 7 | 7 | 7 | 7 | 7 |
|  | ProtestDuration | 1 | 1 | 1 | 1 | 1 | 1 |
|  | nNV | 1 | 1 | 1 | 1 | 1 | 1 |
|  | PeerPressureNumber | 3.3884 | 3.3884 | 3.3884 | 3.3884 | 3.3884 | 3.3884 |
|  | PercentCommitted | 0 | 8.06 | 0 | 0 | 0 | 0 |
|  | PercentImmediateProtest | 0 | 26.4 | 0 | 0 | 0 | 0 |
|  | DefectThreshold | n/a | 0.0547 | n/a | 0.025,0.05,0.1,  0.2,0.3,0.4,0.5 | n/a | n/a |
|  | DefectThresholdStDv | 0 | 0 | 0 | 0 | 0 | 0 |
|  | NVSuccessPercent | n/a | 1 | n/a | n/a | 1,25,50,75,99 | n/a |
|  | PillarProxStrategy | 0 | 0 | 0 | 0 | 0 | 0 |
|  | ActivistSearchVision | 10, unused | 10, unused | 10, unused | 10, unused | 10, unused | 10, unused |
| Run Parameters | R2R_PercentFillActivistMean | 0.8 | 0.8 | 0.8 | 0.8 | 0.8 | 0.8 |
|  | R2R_PercentFillActivistSTD | 0.3 | 0.3 | 0.3 | 0.3 | 0.3 | 0.3 |
|  | R2R_DefectThresholdMin | 0.03 | n/a | 0.03 | n/a | 0.03 | 0.03 |
|  | R2R_DefectThresholdSTD | 0.07 | n/a | 0.07 | n/a | 0.07 | 0.07 |
|  | R2R_DefectThresholdMax | 0.3 | n/a | 0.3 | n/a | 0.3 | 0.3 |
|  | R2R_NVSuccessPercentMin | 1 | n/a | 1 | 1 | n/a | 1 |
|  | R2R_NVSuccessPercentSTD | 16 | n/a | 16 | 16 | n/a | 16 |
|  | R2R_NVSuccessPercentMax | 80 | n/a | 80 | 80 | n/a | 80 |
|  | R2R_PillarProxStrategyPercent | 75 | n/a | n/a | n/a | n/a | 50 |
| Results | Number of Runs | 500 | 500 | 500 | 500 | 500 | 500 |
|  | Participation Size Error | 0.62 | 0.33 | 0.5 | n/a | n/a | n/a |
|  | Probability of Success Error | 2.21 | 15.3 | 9.37 | n/a | n/a | n/a |
|  | Success Percentage | n/a | 69% | 47% | 85%,53%,40%,  31%,23%,5%,  0% | 54%,39%,34%,  27%,21% | n/a |
|  | Case with Most Success | n/a | n/a | n/a | n/a | n/a | n/a |
