# Supplementary material for: A dynamic model of nonviolent resistance strategy
Source: PLoS One. 2022 Jul 27;17(7):e0269976. doi: 10.1371/journal.pone.0269976 (PMC9328538; doi:10.1371/journal.pone.0269976)
Supplement: S4 Table — *Files can be easily modified to run with or without MATLAB Parallel Computing Toolbox, which decreases computation time when used. **Uses MATLAB Statistics and Machine Learning Toolbox. (DOCX) [file pone.0269976.s030.docx]

| **File** | **Purpose** |
| --- | --- |
| ResistanceABMFinal.m | Agent-Based Model |
| SweepMethodFinal.m* | Runs specified parameter cases of ResistanceABMFinal.m |
| EvoMethodFinal.m* | Runs randomly generated cases of ResistanceABMFinal.m, and iterates to optimize matching historic data |
| GetNAVCOData.m | Processes historic data file, NAVCO 1.2 FOR PAPER.xlsx. |
| GetLogisiticRegression.m** | Creates logistic regression of historic data or model data |
| GetRollingAverage.m** | Creates plots used to create peak participation histograms, and although unused for this paper, it can create plots of the probability of success for given peak participation in lieu of the logistic regressions |
| param2mat.m | Used by SweepMethodFinal.m to create a matrix of all cases to run |
| m2instance.m | Used by SweepMethodFinal.m to pick out one parameter case in the matrix created by param2mat.m |
| param4evolution.m | Used by EvoMethodFinal.m to create multiple randomly generated cases with parameters determined by specified mean, standard deviation, and minimum allowable values |
| newparam4evolution.m | Used by EvoMethodFinal.m to create new parameter cases based of previous best case and previously specified standard deviations and minimum allowable values |
| GetParam.m | Creates a vector of a given parameter used in all cases in a vector of parameter structures created by param4evolution.m |
| Data.xls | Historical dataset. |
